# A critical role of hepatic GABA in the metabolic dysfunction and hyperphagia of obesity

**DOI:** 10.1016/j.celrep.2021.109301

**Published:** 2021-06-29

**Authors:** Caroline E. Geisler, Susma Ghimire, Stephanie M. Bruggink, Kendra E. Miller, Savanna N. Weninger, Jason M. Kronenfeld, Jun Yoshino, Samuel Klein, Frank A. Duca, Benjamin J. Renquist

**Affiliations:** 1 School of Animal and Comparative Biomedical Sciences, University of Arizona, Tucson, AZ 85721, USA; 2 Perelman School of Medicine, University of Pennsylvania, Philadelphia, PA 19104, USA; 3 Center for Human Nutrition, Washington University School of Medicine, St. Louis, MO, USA; 4 Lead contact

## Abstract

Hepatic lipid accumulation is a hallmark of type II diabetes (T2D) associated with hyperinsulinemia, insulin resistance, and hyperphagia. Hepatic synthesis of GABA, catalyzed by GABA-transaminase (GABA-T), is upregulated in obese mice. To assess the role of hepatic GABA production in obesity-induced metabolic and energy dysregulation, we treated mice with two pharmacologic GABA-T inhibitors and knocked down hepatic GABA-T expression using an antisense oligonucleotide. Hepatic GABA-T inhibition and knockdown decreased basal hyperinsulinemia and hyperglycemia and improved glucose intolerance. GABA-T knockdown improved insulin sensitivity assessed by hyperinsulinemic-euglycemic clamps in obese mice. Hepatic GABA-T knockdown also decreased food intake and induced weight loss without altering energy expenditure in obese mice. Data from people with obesity support the notion that hepatic GABA production and transport are associated with serum insulin, homeostatic model assessment for insulin resistance (HOMA-IR), T2D, and BMI. These results support a key role for hepatocyte GABA production in the dysfunctional glucoregulation and feeding behavior associated with obesity.

## In brief

Geisler et al. show that GABA-transaminase catalyzes GABA synthesis in the liver of obese mice, resulting in hyperinsulinemia, insulin resistance, and hyperphagia. In people with obesity, liver GABA-transaminase expression is positively associated with hyperinsulinemia. Thus, GABA-transaminase inhibitors may be effective at restoring glucose homeostasis in obese, hyperinsulinemic, insulin-resistant individuals.

## Graphical Abstract

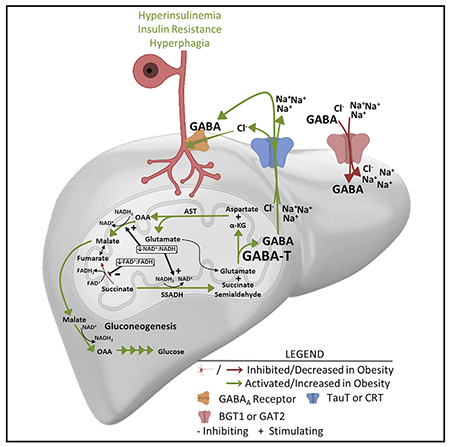

## INTRODUCTION

Type II diabetes (T2D) affects 30 million Americans, and an additional 81 million Americans have pre-diabetes (Centers for Disease Control and Prevention, 2017). Thus, 46% of the US adult population is affected by diabetes, the 7^th^ leading cause of death in America, which consumes 1 in every 7 US health care dollars (American Diabetes Association, 2018). The high prevalence, mortality, and economic burden of T2D underscores a critical need for the development of additional therapeutics to treat diabetes.

Hepatic steatosis is a hallmark of T2D, as accumulation of hepatic lipids strongly correlates with the severity of peripheral insulin resistance and hyperinsulinemia (Chang et al., 2013; Chon et al., 2016; Kotronen et al., 2008). Furthermore, 87% of diabetics are overweight or obese (Centers for Disease Control and Prevention, 2017). Obesity and T2D are also characterized by dysregulated energy homeostasis, particularly diminished meal-induced satiety, which can result in excessive energy intake (Erdmann et al., 2005; Knudsen et al., 2014; Timper and Brüning, 2017). Interestingly, hepatic lipid accumulation is directly associated with increased energy intake. In individuals with non-alcoholic fatty liver disease (NAFLD), the percent hepatic steatosis positively correlates with total energy intake and carbohydrate intake (Gonzalez et al., 2013). These findings suggest the possibility that hepatic lipid accumulation may drive obesity-induced hyperinsulinemia, insulin resistance, and hyperphagia.

The liver produces and releases hepatokines into circulation in response to acute and chronic nutrient status (Meex and Watt, 2017). For example, FGF21 and ANGPTL4 are secreted from hepatocytes in response to liver nutrient flux and can act in an endocrine fashion to impact whole-body metabolism. In line with this, we have recently established that obesity-induced hepatic lipid accumulation increases hepatocyte production and release of the inhibitory neurotransmitter, GABA, in mice (Figure 4A in Geisler et al., 2021 [this issue of *Cell Reports*]) that we hypothesize acts in a paracrine fashion to decrease the firing activity of the hepatic vagal afferent nerve (HVAN) to regulate insulin secretion and sensitivity (Figure 5 in Geisler et al., 2021). These findings provide a mechanistic link explaining how hepatic lipid accumulation, though increasing extracellular hepatocyte-produced GABA to impair HVAN signaling, drives the development of metabolic diseases.

The GABA shunt classically refers to a tricarboxylic acid cycle detour that converts α-ketoglutarate to succinate and concomitantly breaks down a molecule of GABA via GABA-transaminase (GABA-T) (Hassel et al., 1998). However, in the liver, GABA-T mediates GABA synthesis (White, 1981). We have proposed that hepatic lipids activate reversed GABA shunt activity in hepatocytes and that hepatic GABA and glucose production are metabolically linked (Figure 5 in Geisler et al., 2021). It remains completely untested whether manipulating this GABA shunt can prevent hepatic-steatosis-derived metabolic disease. Thus, GABA-T represents a promising target to decrease hyperinsulinemia and insulin resistance by limiting hepatic GABA production. Accordingly, in the current manuscript, we employed two models to limit hepatic GABA production: (1) pharmacologic inhibition of GABA-T activity and (2) antisense oligonucleotide (ASO)-mediated knockdown of hepatic-specific GABA-T expression. Using these models, we assessed systemic glucose homeostasis to examine the causative role between obesity-induced hepatic GABA production and obesity-induced hyperinsulinemia and insulin resistance. We also assessed food intake and energy expenditure to understand the role of hepatic GABA production in the dysregulation of energy homeostasis in obesity.

## RESULTS

### GABA-T inhibition improves glucose homeostasis in obesity

**Figure 1.**
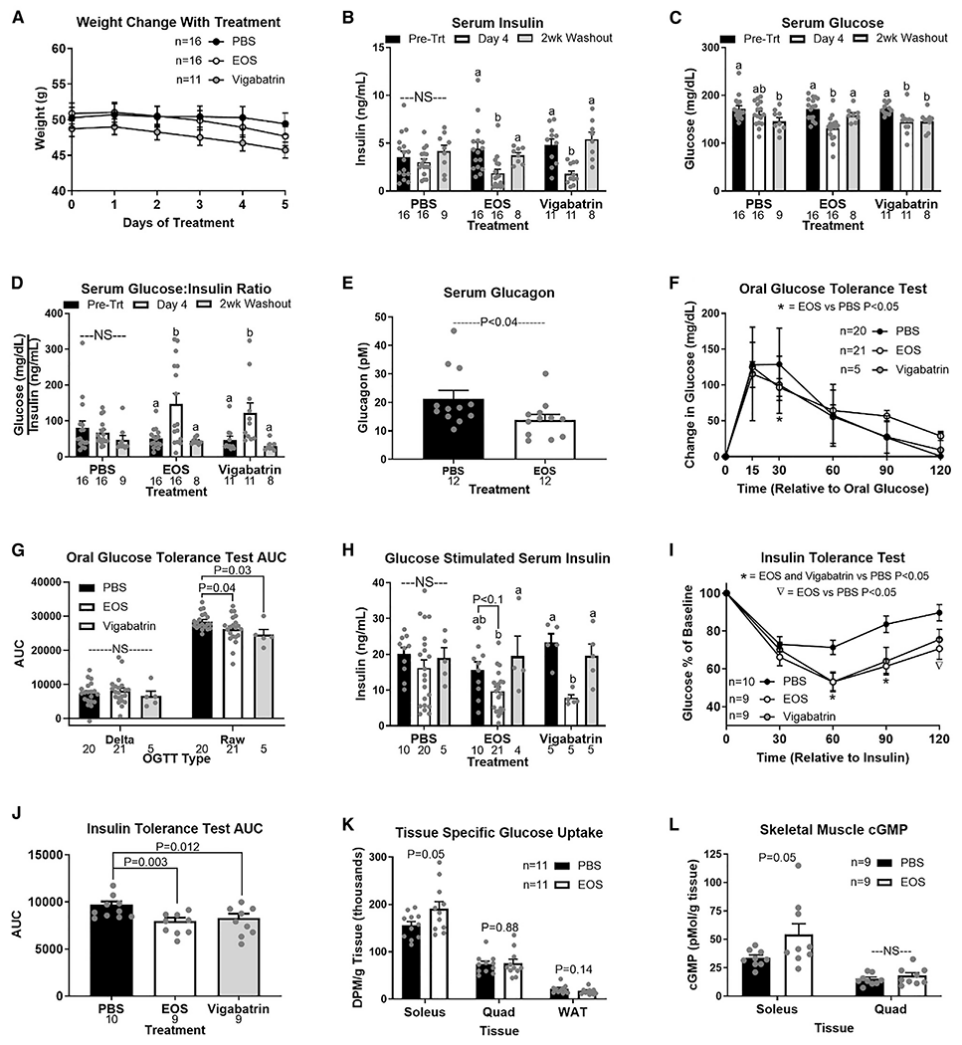
GABA-transaminase inhibition improves glucose homeostasis in obesity HFD-induced obese mice were treated with GABA-transaminase inhibitors ethanolamine-O-sulfate (EOS) or vigabatrin (8 mg/day) or phosphate-buffered saline (PBS) (control) for 5 days. (A and B) Bars that do not share a common letter differ significantly within injection treatment (p < 0.05; number below bar denotes n per group) (A) Body weight during treatment. (B–D) Basal serum insulin (B), glucose (C), and glucose:insulin ratio (D) pre-treatment, on treatment day 4, and after a 2-week washout. (E) Serum glucagon in response to EOS. (F and G) Oral glucose tolerance (OGTT) (F) and OGTT area under the curve (AUC) (G) on treatment day 4. (H) Glucose-stimulated serum insulin pre-treatment, on treatment day 4, and after a 2-week washout. (I and J) Insulin tolerance test (ITT) (I) and ITT AUC (J) on treatment day 4. (K and L) Tissue-specific ^3^H-2-deoxy-D-glucose (10 μCi/mouse; K) uptake and cGMP content (L) on treatment day 5. DPM, disintegrations per minute; NS, non-significant. All data are presented as mean ± SEM.

To directly assess the role of GABA-T in obesity-induced metabolic dysfunction, we treated high-fat-diet (HFD)-induced obese mice with one of two irreversible GABA-T inhibitors, ethanolamine-O-sulfate (EOS) or vigabatrin (8 mg/day intraperitoneally [i.p.]) or phosphate-buffered saline vehicle. Both inhibitors reduce hepatic GABA-T activity by over 90% within 2 days (Qume and Fowler, 1996). Through 5 days of treatment, body weight remained similar among EOS-, vigabatrin-, and saline-injected mice (Figure 1A). 4 days of EOS or vigabatrin treatment decreased serum insulin and glucose concentrations and increased the glucose:insulin ratio relative to pre-treatment (Figures 1B–1D). 2-week washout from EOS or vigabatrin resulted in a return of serum insulin and the glucose:insulin ratio to pre-treatment levels (Figures 1B–1D). EOS treatment (5 days) decreased serum glucagon relative to control mice (Figure 1E). Blood glucose concentrations during an oral glucose tolerance test (OGTT) (raw area under the curve [AUC]), but not relative glucose clearance (delta AUC) was improved by 4 days of GABA-T inhibition (Figures 1F and 1G). Coincident with this improved clearance, glucose-stimulated serum insulin was decreased by vigabatrin and tended to be decreased by EOS relative to pre-treatment concentrations (Figure 1H). 2-week washout from EOS and vigabatrin markedly increased glucose-stimulated serum insulin (Figure 1H). Both GABA-T inhibitors improved insulin sensitivity assessed by insulin tolerance test (ITT) within 4 days of initiating treatment (Figures 1I and 1J). As oral drug delivery is preferred in clinic, we conducted another series of studies with EOS provided in the drinking water (3 g/L) for 4 days. EOS in the drinking water similarly improved measures of glucose homeostasis, and the response washed out after 2 weeks without EOS in the drinking water (Figure S1).

To further assess the mechanism by which GABA-T inhibition improves insulin-stimulated glucose clearance, we measured tissue-specific ^3^H-2-deoxy-D-glucose (2DG) uptake following an oral glucose gavage on day 5 of EOS or saline treatment. EOS treatment increased 2DG uptake by the soleus (22%) but did not affect 2DG clearance by the quadriceps femoris (quad) or gonadal white adipose tissue (WAT) (Figure 1K). Given that blood perfusion is a key regulator of insulin action and glucose clearance, we subsequently measured cyclic guanosine monophosphate (cGMP), a key second messenger downstream of nitric oxide (NO) signaling that regulates blood flow (Archer et al., 1994). EOS increased cGMP in the soleus (59%) but had no effect in quad (Figure 1L).

In lean mice, which have low hepatic GABA production (Figures 4A and 4B in Geisler et al., 2021), there was no effect of EOS or vigabatrin (8 mg/day i.p.) on serum insulin concentrations or insulin sensitivity (Figures S1I, S1O, and S1P). In lean mice, GABA-T inhibition decreased glucose-stimulated serum insulin (Figure S1N).

### Hepatic GABA-T knockdown improves obesity-induced metabolic dysfunction

**Figure 2.**
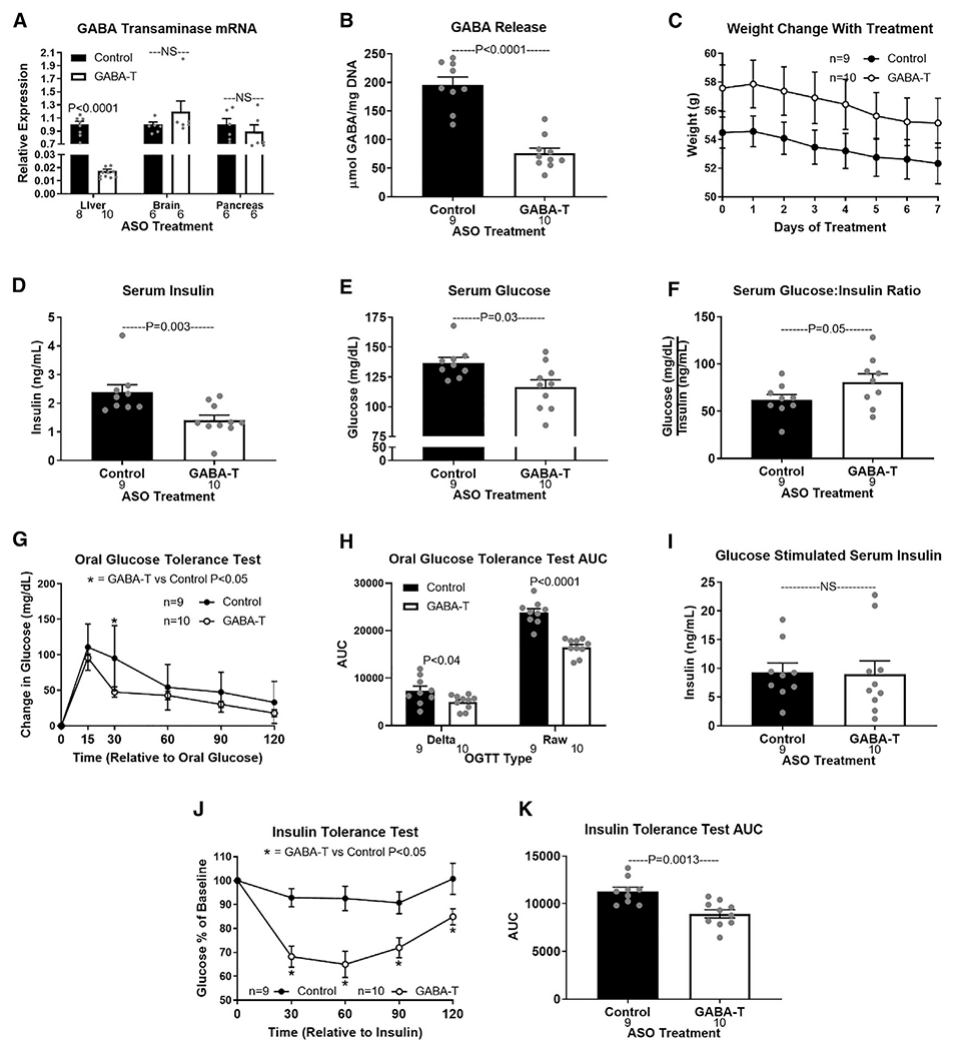
Acute hepatic GABA-transaminase knockdown improves obesity-induced metabolic dysfunction (A) GABA-T mRNA expression in liver, whole brain, and pancreas after 1 week of injections with a GABA-T targeted or scramble control antisense oligonucleotide (ASO) (12.5 mg/kg i.p. twice weekly) in high-fat-diet-induced obese mice. (B) Release of GABA (μmol/mg DNA) from hepatic slices. (C) Body weight during treatment. (D–F) Basal serum insulin (D), glucose (E), and glucose:insulin ratio (F). (G–K) OGTT (G), OGTT AUC (H), oral glucose-stimulated serum insulin (I), ITT (J), and ITT AUC (K). Number below bar denotes n per group. All data are presented as mean ± SEM.

To overcome the limitation of global pharmacologic inhibitors, we next used an ASO model to specifically knockdown hepatic GABA-T expression. Peripherally administered ASOs do not cross the blood brain barrier (Jaeger and Banks, 2005). Outside the central nervous system, the liver and pancreas express the most GABA-T (Uhlén et al., 2015). A GABA-T targeted ASO (12.5 mg/kg i.p. twice weekly) decreased hepatic GABA-T mRNA expression by >98% within 1 week. Importantly, this GABA-T-targeted ASO did not affect pancreatic or whole-brain GABA-T mRNA expression (Figure 2A). To establish the key role of GABA-T in liver slice GABA production, we measured *ex vivo* liver slice GABA release. GABA-T knockdown cut obesity-induced liver slice GABA release by 61% (Figure 2B). 1 week of GABA-T knockdown in obese mice did not affect body weight but decreased basal serum insulin and glucose concentrations and elevated the glucose:insulin ratio (Figures 2C–2F). GABA-T-targeted ASO injections also improved oral glucose clearance without affecting oral glucose stimulated serum insulin concentrations (Figures 2G–2I) and improved insulin sensitivity compared to scramble control ASO-injected mice (Figures 2J and 2K). These findings directly implicate hepatic GABA production in the development of obesity-induced hyperinsulinemia and insulin resistance.

Compared with control ASO, chronic (4-week) GABA-T ASO treatment decreased *ex vivo* liver slice GABA release and decreased body mass and serum insulin while increasing the glucose:insulin ratio (Figures S2A–S2C and S2E). Thus, the metabolic response to GABA-T knockdown persists. Admittedly, a decrease in body mass with chronic ASO treatment could contribute to the improvements in glucose homeostasis.

**Figure 3.**
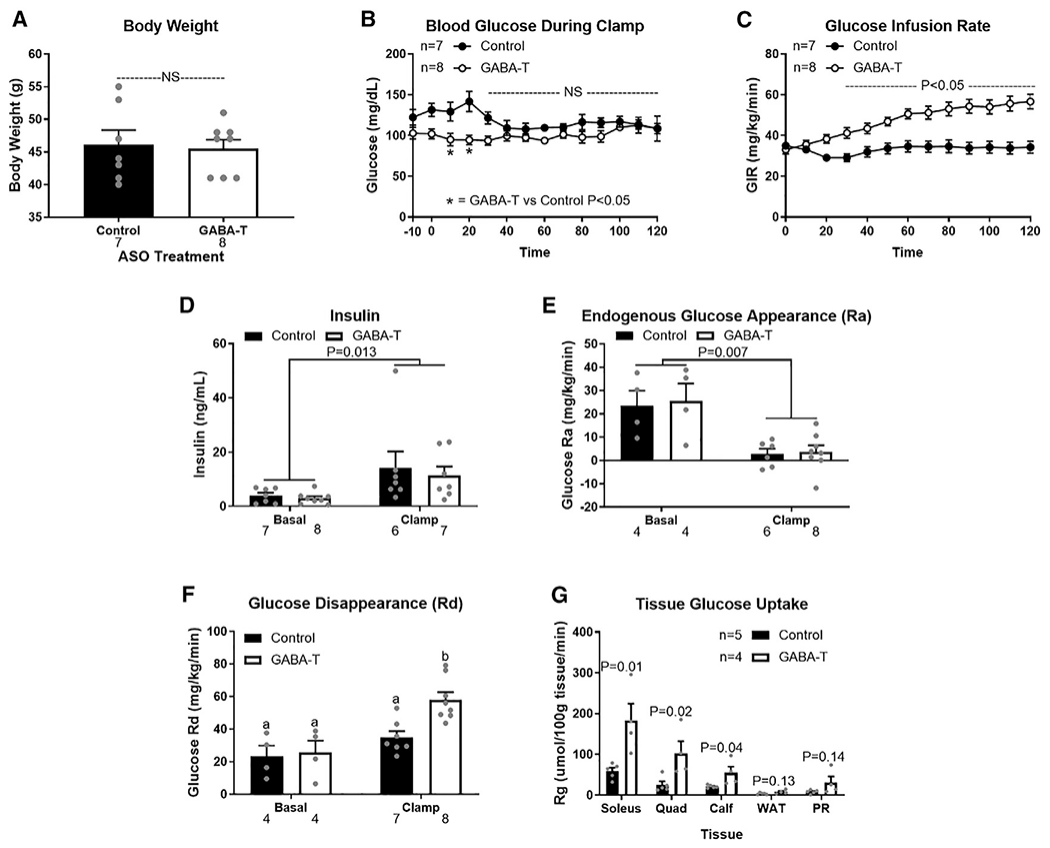
1 week of hepatic GABA-transaminase knockdown improves insulin sensitivity assessed by hyperinsulinemic euglycemic clamp High-fat-diet-induced obese mice received 1 week of injections with a GABA-T targeted or scramble control ASO (12.5 mg/kg i.p. twice weekly) before hyperinsulinemic euglycemic clamps were performed. (A) Body weight the day of clamp procedures. (B and C) Blood glucose concentrations (B) and glucose infusion rate during the clamps (C). (D) Serum insulin concentrations before insulin infusion (basal) and during the clamp. (E and F) Endogenous glucose appearance (Ra) (E) and glucose disappearance (Rd) (F) before insulin infusion (basal) and during the clamp. (G) Tissue specific ^14^C-2-deoxyglucose uptake (PR, perirenal adipose tissue; WAT, white adipose tissue). Number below bar denotes n per group. All data are presented as mean ± SEM.

To assess the effect of GABA-T knockdown on insulin action before body mass is affected, we performed hyperinsulinemic-euglycemic clamps in diet-induced obese mice treated with scramble control or GABA-T-targeted ASOs for 1 week. Body weight on the day of clamp was not affected by treatment (Figure 3A). All time points are relative to the onset of insulin (4 mU/kg/min) and variable rate 3-[^3^H]-D-glucose in dextrose infusion at time 0, with the basal period referencing pre-0 measurements. Blood glucose concentrations were lower in GABA-T ASO-treated mice during the first 20 min of the clamp, but not different between ASO treatments from 30 to 120 min of the clamp, during which euglycemia was achieved in both groups (Figure 3B). To maintain the same level of euglycemia, GABA-T knockdown mice required a higher glucose infusion rate (GIR) (mg/kg/min), which was significant from 30 to 120 min of the clamp (Figure 3C). Plasma insulin concentrations did not differ by ASO treatment during either the basal period or during the clamp (Figure 3D). Importantly, insulin concentrations were elevated during the clamp (Figure 3D; p = 0.013). GABA-T knockdown did not affect the rate of endogenous glucose appearance (Ra) during the basal or clamp periods (Figure 3E). The basal rate of glucose disappearance (Rd) did not differ between ASO treatments. However, hyperinsulinemia during the clamp nearly doubled Rd in GABA-T knockdown, but not control, mice (Figure 3F). After 120 min of the clamp, mice received a bolus of ^14^C-2-dexyglucose and were sacrificed 30 min later to assess tissue-specific glucose uptake. GABA-T knockdown improved glucose uptake by the soleus, quadricep, and gastrocnemius skeletal muscles (Figure 3G).

**Figure 4.**
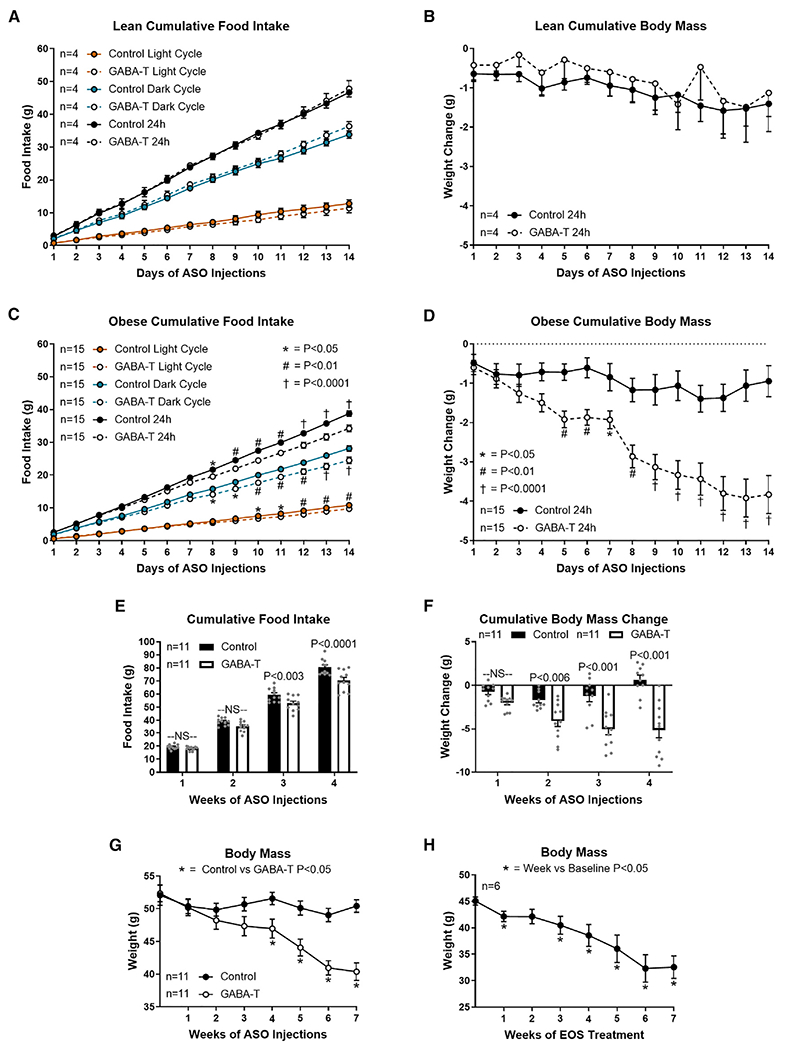
Obesity-induced hepatic GABA production increases phagic drive Cumulative food intake and body mass during the first 2 weeks of GABA-T targeted or scramble control ASO injections (12.5 mg/kg i.p. twice weekly) in lean (A and B) and diet-induced obese (C–G) mice. Cumulative light cycle, dark cycle, and daily food intake (A) and cumulative body mass change (B) in lean mice are shown. Cumulative light cycle, dark cycle, and daily food intake (C) and cumulative body mass change (D) in obese mice are shown. Weekly cumulative food intake (E), cumulative body weight change (F), and body mass (G) during ASO treatment are shown. Body mass during chronic EOS treatment is shown (3 g/L in drinking water). All data are presented as mean ± SEM.

In chow-fed mice, hepatic GABA production is limited (Figures 4A and 4B in Geisler et al., 2021). In turn, we expected minimal responses to inhibition of the GABA shunt in lean mice. We tested two GABA-T-targeted ASOs that both decreased hepatic GABA-T mRNA expression by ≥80% within 1 week and ≥94% after 4 weeks of treatment. Neither altered pancreatic or whole-brain GABA-T mRNA expression (Figures S3A and S3B). Chronic (4-week) GABA-T knockdown did not affect body weight or glucoregulatory measures in lean mice (Figures S3C–S3K). This agrees with our observations that acute GABA-T inhibition with EOS does not alter glucose homeostasis in lean mice (Figures S1I–S1P). Instead, beneficial effects of GABA-T inhibition are specific to obesity, due to an increase in the GABA shunt activity and subsequent hepatic GABA production.

### The hepatic vagal nerve is essential to improvements in glucose homeostasis resulting from GABA-T inhibition or knockdown

Hepatic vagotomy prevents the liver from altering afferent signaling to the brain, without affecting normal vagal afferent input originating from cell bodies in the nodose ganglia (Peters et al., 2013). In turn, we expect that vagotomy prevents inhibition of the vagal afferent tone by GABA produced in the liver. We hypothesize that hepatic GABA mediates the effects that we have reported by acting on the HVAN to induce hyperinsulinemia and insulin resistance. Accordingly, we assessed the effect of EOS treatment in HFD-induced obese hepatic vagotomized and sham-operated mice. Although body weight did not differ between surgical groups during EOS treatment (Figure S4A), the response to EOS was dependent on an intact hepatic vagal nerve. In sham-operated mice, EOS decreased serum insulin and glucose, elevated the glucose:insulin ratio, improved oral glucose tolerance, diminished glucose-stimulated serum insulin concentrations, and tended to improve insulin sensitivity as measured by an ITT (Figures S4B and S4K). Washout restored all parameters to pre-treatment measures. Vagotomy eliminated most of the responses to EOS (Figures S4B–S4L). Because we propose that hepatic GABA production supports gluconeogenic flux (Figure 5 in Geisler et al., 2021), we expected GABA-T inhibition to decrease gluconeogenesis through direct actions at the liver. In turn, diminished hepatic glucose output explains the vagal nerve independent decrease in serum glucose during EOS treatment in hepatic vagotomized mice (Figure S4C).

We next assessed the effect of 4 weeks of GABA-T knockdown (12.5 mg/kg GABA-T-targeted ASO i.p. twice weekly) in diet-induced, obese, sham-operated and hepatic vagotomized mice. Consistent with previous observations (Figure 1B in Geisler et al., 2021), pre-treatment body weight was lower in obese, vagotomized mice compared to sham-operated control mice (Figure S2G). GABA-T knockdown decreased basal serum insulin in sham, but not in vagotomized, mice (Figure S6B). As shown with pharmacological GABA-T inhibition (EOS), GABA-T knockdown decreased basal serum glucose concentrations independent of the hepatic vagal nerve (Figure S2I), again likely due to reduced hepatic glucose output. After 4 weeks of GABA-T ASO injections, serum glucagon oral glucose tolerance, glucose-stimulated serum insulin, and insulin sensitivity were not different between surgical treatments (Figures S2K–S2P). Hepatic vagotomy protects against obesity-induced glucose intolerance and insulin resistance (Figure 1 in Geisler et al., 2021). Here, we show that GABA-T knockdown allowed sham-operated animals to achieve similar glucose tolerance, glucose-stimulated serum insulin, and insulin sensitivity to that measured in hepatic vagotomized mice.

Hepatic vagotomy lowers serum insulin, improves glucose tolerance, and improves insulin sensitivity, recapitulating the response to GABA-T ASO-mediated knockdown or GABA-T pharmacological inhibition. This further supports our hypothesis that an obesity-induced increase in hepatic GABA production relies upon the HVAN to induce hyperinsulinemia and insulin resistance.

### GABA-T knockdown decreases fat mass by decreasing food intake without affecting energy expenditure in obesity

Given that hepatic GABA-T knockdown for 4 weeks decreased body weight in obese mice (Figure S2B), we hypothesized that GABA-T knockdown may decrease food intake or increase energy expenditure. Accordingly, we assessed daily food intake and body mass during the light and dark cycle for the first 2 weeks of ASO treatment in lean and obese mice. In lean mice, GABA-T knockdown did not affect cumulative light cycle, dark cycle, or daily food intake or daily body mass change (Figures 4A and 4B). In obese mice, GABA-T knockdown decreased cumulative light cycle, dark cycle, and daily food intake (Figure 4C). To ensure that the metabolic changes were not merely a result of this decrease in food intake, we pair-fed mice to match the decrease in food consumption associated with GABA-T knockdown (Figure S5B). This 6.57% ± 0.34% dietary restriction did not affect the change in glucose during an OGTT or insulin sensitivity (Figures S5C–S5F). Cumulative weekly food intake remained lower through 4 weeks of GABA-T knockdown (Figure 4E). This suppression of food intake was accompanied by a decrease in body mass in GABA-T ASO-treated mice (Figures 4D and 4F). In fact, GABA-T knockdown continued to decrease body weight during 7 weeks of treatment (Figure 4G). Similarly, 7 weeks of continuous exposure to EOS in the drinking water (3 g/L) dramatically decreased body mass in diet-induced obese mice (Figure 4H). These data suggest that sensitivity to the weight loss effect of limiting hepatocyte GABA production is maintained throughout treatment but is less significant as body mass returns to normal.

Interestingly, GABA-T knockdown did not alter food intake in response to a 16-h fast in either chow-fed lean or HFD-induced obese mice (Figures S6A and S6B). This proposes that hepatic GABA signals to regulate *ad libitum* light and dark cycle food intake without affecting the fasting-induced drive for refeeding. Accordingly, GABA-T knockdown did not affect fasting mRNA expression of the canonical hypothalamic neuropeptides regulating food intake (neuropeptide Y [NPY], agouti-related peptide [AgRP], and pro-opiomelanocortin [POMC]; Figure S6C). Admittedly, we cannot rule out an effect of central GABA-T knockdown, as we did observe a 14% reduction in hypothalamic GABA-T expression at 7 weeks of GABA-T ASO injections (Figure S6C). 4 weeks of GABA-T ASO treatment did not affect either hypothalamic or whole-brain GABA-T mRNA expression (Figure S6D). Although ASOs do not cross the blood brain, it is possible that the ASO altered food intake or metabolic function by acting on circumventricular areas in the brain. Although we have not investigated the possible involvement of circumventricular GABA-T knockdown, data from the hepatic vagotomy model would suggest that the hepatic vagal nerve is involved (Figure S8B).

The anorexigenic hormone leptin induces satiety and weight loss, whereas leptin resistance in obesity contributes to hyperphagia and weight gain (Frederich et al., 1995; Halaas et al., 1995). We tested whether GABA-T knockdown in obesity may have improved leptin sensitivity as a potential mechanism to decrease appetite and cause weight loss. A single 6AM injection of leptin (2 mg/kg) did not affect food intake in either obese GABA-T or control ASO-treated mice at any time point, suggesting that all mice were leptin resistant (Figure S6E). Consistent with the decreased food intake previously observed in response to GABA-T knockdown, we again saw that GABA-T knockdown decreased light cycle, dark cycle, and 24-h food intake independent of injection (Figure S6E). Relative weight change was also not affected by leptin in control or GABA-T ASO obese mice (Figure S6F). Thus, enhanced leptin sensitivity is not mediating the diminished phagic drive in response to GABA-T knockdown in obesity. A single 6AM leptin injection decreased light cycle and 24-h food intake in chow-fed mice without affecting body weight (Figures S6G and S6H).

We next investigated whether altered energy expenditure contributed to the body mass loss with GABA-T knockdown in obesity. Energy expenditure (kcal/h), assessed by respiration calorimetry and corrected using the covariate of lean body mass, was not altered by 1 or 4 weeks of GABA-T knockdown (Figures S7A–S7C). In addition, there was no effect of GABA-T knockdown on the composition of oxidized macronutrients (respiratory exchange ratio [RER]), daily water intake, or daily activity (Figures S7D–S7I). Thus, the effects of GABA-T knockdown on body mass appear independent of energy expenditure or nutrient oxidation.

**Figure 5.**
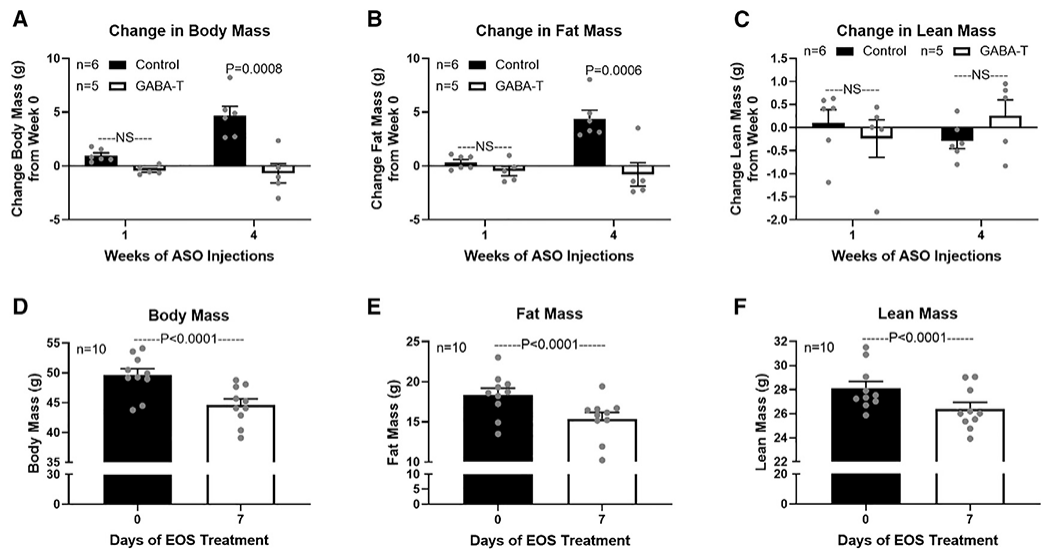
GABA-transaminase knockdown or inhibition decreases body mass and fat mass Body composition in ASO-treated mice was assessed by dual-energy X-ray absorptiometry (DEXA) at the UCDavis Mouse Metabolic Phenotyping Center. Body composition in ethanolamine-O-sulfate (EOS)-treated mice was assessed by EchoMRI 900 at The University of Arizona. Change in body mass (A), fat mass (B), and lean mass (C) after 1 and 4 weeks of GABA-T targeted or scramble control ASO (12.5 mg/kg i.p. twice weekly) relative to pre-treatment body composition are shown. Body mass (D), fat mass (E), and lean mass (F) on day 0 and 7 of EOS treatment are shown (3 g/L in drinking water). All data are presented as mean ± SEM.

Body composition, assessed by dual-energy X-ray absorptiometry (DEXA), revealed that 4 weeks of GABA-T knockdown decreased total body mass and fat mass without affecting lean mass relative to control mice (Figures 5A–5C). The decreased fat mass with GABA-T knockdown suggests that weight loss induced by limiting hepatocyte GABA production reflects a loss of adiposity. We additionally assessed body composition on day 0 and 7 of providing obese mice with EOS in their drinking water (3 g/L). We found that EOS treatment decreased total body mass (10%), fat mass (16.27%), and lean mass (6.15%). Although there was a small loss of lean mass, diminished fat mass constitutes most of the lost body mass with EOS treatment.

### Hepatic vagotomy decreases light cycle food intake on HFD. GABA-T knockdown normalizes food intake in sham mice to that observed in vagotomized mice

We had previously reported that hepatic vagotomy decreased weight gain on a HFD (Figure 1B in Geisler et al., 2021). Here, we show that hepatic vagotomy decreases 1-week cumulative light cycle food intake by 22% in diet-induced obese mice, resulting in a 5.3% decrease in cumulative 24-h food intake (Figure S8A). We continued daily food intake measurements for the next 2 weeks as all mice were treated with the GABA-T-targeted ASO. In response to GABA-T knockdown, the previously observed difference in food intake in sham-operated and vagotomized mice was eliminated (Figures S8B and S8C). The GABA-T ASO resulted in a greater cumulative week 4 body mass loss in sham-operated than in vagotomized mice, which experienced no net change in body mass (Figure S8D). Thus, the glucoregulatory, phagic, and body mass changes in response to GABA-T knockdown all appear to be dependent on the HVAN. These data support that hepatic vagotomy eliminates the GABA-induced decrease in HVAN activity from being communicated to the brain and accordingly protects against obesity-induced perturbations of glucose and energy homeostasis.

### Associations between hepatic GABA system and glucoregulatory markers in humans with obesity

**Figure 6.**
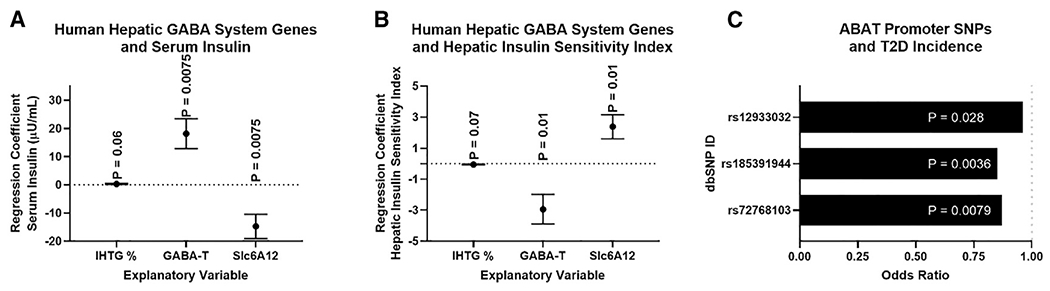
Associations between hepatic GABA system and glucoregulatory markers in people with obesity Multivariate regressions, including intrahepatic triglyceride % (IHTG%), hepatic *ABAT* (GABA-T) mRNA, and the hepatic GABA transporter (*SLC6A12*) mRNA as explanatory variables for variations in serum insulin (A) or hepatic insulin sensitivity index (HISI) (B), *ABAT* and *SLC6A12* mRNA (FPKMUQ; fragments per kilobase million reads upper quartile) were quantified by RNA sequencing (RNA-seq) from liver tissue. Single-nucleotide polymorphisms (SNPs) in the *ABAT* promoter are associated with a decreased risk of type 2 diabetes (T2D) (C). All data are presented as mean ± SEM.

To understand the potential clinical relevance of these findings, we assessed the hepatic mRNA expression of *ABAT* (encodes for GABA-T) and the 4 GABA transporters (*SLC6A6*, encodes for taurine transporter [TauT]; *SLC6A8*, encodes for the creatine transporter [CRT]; *SLC6A12*, encodes for the betaine-GABA transporter 1 [BGT1]; and *SLC6A13*, encodes for GABA transporter 2 [GAT2]) in 19 people with obesity (age 45 ± 3 years old; 2 men and 17 women), who were carefully characterized by measuring intrahepatic triglyceride (IHTG) content using magnetic resonance imaging (MRI) and insulin sensitivity using the hyperinsulinemic-euglycemic clamp procedure (HECP) in conjunction with stable isotopically labeled glucose tracer infusion. The subjects had a wide range in IHTG content, plasma insulin concentration, and measures of insulin sensitivity (Table S1). There was a trend toward a positive correlation between IHTG % and basal plasma insulin concentration (p = 0.06; Figure 6A) and a negative correlation between IHTG % and hepatic insulin sensitivity index (HISI) (a product of the basal endogenous glucose production rate and plasma insulin concentration; p = 0.07; Figure 6B; Table S2).

Hepatic *ABAT* mRNA expression was positively associated with plasma insulin and negatively with HISI (Figures 6A and 6B). Using the Accelerating Medicines Partnership Type 2 Diabetes Knowledge Portal database, we identified that single-nucleotide polymorphisms (SNPs) in the reporter region of *ABAT* are associated with decreased risk of T2D (Figure 6C; Table S3). Importantly, we found no promoter region SNPs that were significantly associated with an increased odds ratio for T2D. Moreover, given the key role of GABA-T in the central nervous system, it is not surprising that SNPs in the *ABAT* gene were not identified. These data support the notion that GABA-T is a driver of insulin resistance in people.

By using our *ex vivo* liver slice model, we have shown that pharmacologically inhibiting BGT1 increased media GABA content, proposing that, although this transporter is bidirectional, flux is more heavily driven toward GABA import (Figure 5 in Geisler et al., 2021). Moreover, missense mutations in *SLC6A12* are associated with an increased risk of T2D. In fact, a premature stop codon is associated with a 16-fold increase in the risk for T2D (Table S3 in Geisleret al., 2021). Here, we show that hepatic *SLC6A12* mRNA is negatively associated with plasma insulin concentrations and positively associated with HISI (Figures 6A and 6B). Thus, we hypothesize that *SLC6A12* expression is primarily indicative of GABA re-uptake into hepatocytes, which depresses GABA signaling onto the HVAN.

Because we have established that GABA-T knockdown and inhibition reduces food intake and body weight, we utilized the knowledge portal diabetes database to identify SNPs associated with BMI. We found missense mutations in *SLC6A12* (6), *SLC6A6* (3), and *SLC6A13* (4) that were associated with increased BMI (Table S4). Taken together, the data obtained from the studies conducted in people support the potential clinical translation of our findings in the mouse.

## DISCUSSION

The liver signals in an endocrine fashion through a myriad of hepatokines and metabolites that change with the degree of hepatic steatosis to reflect altered metabolic homeostasis (Holland et al., 2017; Meex and Watt, 2017; Petersen et al., 2016). We report liver-produced GABA is a steatosis-affected hepatokine that alters glucose homeostasis. Unlike other hepatokines, which act in an endocrine fashion, our findings suggest GABA is acting locally in a paracrine fashion on the parasympathetic nervous system to mediate its downstream metabolic effects. Muting hepatic GABA production by pharmacologic inhibition or ASO-mediated knockdown of GABA-T attenuates obesity-induced hyperinsulinemia and insulin resistance. Beyond the improvements in glucose homeostasis, hepatic GABA-T knockdown decreases food intake, causing a decrease in adiposity and weight loss.

### Glucose homeostasis

Preventing hepatic GABA production improves insulin sensitivity primarily by increasing skeletal muscle glucose clearance (Figures 1K and 3G). Our results propose that some of the improvements in glucose clearance may be mediated by increased blood flow. Vasodilation of the microvasculature accounts for 40% of insulin-stimulated muscle glucose uptake (Vincent et al., 2003). Insulin and acetylcholine signaling at endothelial cells stimulates NO production, which signals to smooth muscle cells inducing cGMP production and vasodilation (Archer et al., 1994; Gericke et al., 2011). The microvascular vasodilatory response is reduced in obesity, directly contributing to systemic insulin resistance (Cleland et al., 1999; Indumathy et al., 2015; Van Guilder et al., 2006). We showed that EOS treatment increased soleus muscle cGMP concentrations (Figure 1L). Future work will focus on understanding how hepatic GABA signaling at the HVAN regulates endothelial NO production and muscle perfusion.

### Food intake regulation

Although weight loss itself improves insulin sensitivity and metabolic health (Schenk et al., 2009), GABA-T knockdown improves glucose homeostasis independent of the decrease in food intake and body weight. 1 week of GABA-T ASO treatment does not decrease food intake (Figure 4E) or body weight (Figures 2C and 4F) compared to controls, yet basal serum insulin concentrations, glucose tolerance, and insulin sensitivity are markedly improved (Figures 2D, 2G, and 2J). Moreover, pair feeding to match the hypophagia caused by the GABA-T ASO did not improve glucose clearance or insulin sensitivity (Figures S5C–S5F).

The HVAN has long been implicated in transmitting liver-derived signals to the hindbrain to regulate feeding behavior (Langhans et al., 1985; Russek, 1963). Early work established that hepatic portal infusion of glucose, amino acids, and lipids suppresses food intake (Niijima and Meguid, 1995; Randich et al., 2001; Shurlock and Forbes, 1984; Tordoff and Friedman, 1986), although more recent studies support that carbohydrate signals originating from the liver regulate feeding behavior through HVAN-dependent mechanisms (López-Soldado et al., 2017; Visinoni et al., 2012). Peripheral satiation factors, including glucagon-like peptide-1 (GLP-1), cholecystokinin (CCK), lipids, and leptin, reduce food intake dependent upon increasing gastric and hepatic vagal branch afferent firing, although the orexigenic hormone ghrelin suppresses vagal afferent tone (Cox and Randich, 1997; Date et al., 2002; Krieger et al., 2016; Niijima, 2011; Peters et al., 2006; Randich et al., 2001). We propose an addition to this regulation of vagal nerve activity by metabolites and gut hormones, suggesting that hepatic lipid accumulation stimulates hepatic GABA release to depress HVAN activity. Consistent with the effects of other appetite regulators, GABA-mediated suppression of HVAN activity would be expected to increase phagic drive; removal of this inhibitory signal would be expected to increase HVAN firing and reduce food intake. In lean mice, which have low levels of hepatic GABA release, GABA-T knockdown did not alter food intake. Thus, hepatocyte-released GABA represents an orexigenic signal enhanced by obesity.

Diet-induced obesity dysregulates the diurnal pattern of food intake. Mice on a chow diet eat ~20% of their daily food intake during the light cycle; this increases to ~30% with HFD feeding (Kohsaka et al., 2007). In healthy mice, vagal afferent receptor populations are regulated by nutritional state, expressing an orexigenic profile during fasting and an anorexigenic profile upon refeeding (Dockray, 2014). In turn, in chow-fed mice, anorexigenic mechano-stimuli more effectively stimulate gastric vagal afferent nerve activity during the light cycle (Kentish et al., 2013). These circadian patterns are lost with diet-induced obesity (Dockray, 2014; Kentish et al., 2016). Together, these changes promote increased food intake during the light cycle and support the hypothesis that the dysregulation of feeding behavior in obesity is partially mediated by aberrant vagal signaling.

In lean mice, hepatic vagotomy shifts the diurnal feeding pattern to increase light cycle food intake (Hepler et al., 2016; Louis-Sylvestre, 1978), potentially mediated by the loss of peripheral light cycle anorexigenic stimuli. We report that diet-induced obese, hepatic vagotomized mice eat less during the light cycle than sham-operated controls, suggesting that hepatic vagotomy protects against the obesity-induced increase in daytime feeding. We hypothesize that preventing the GABA-mediated depression of HVAN activity from reaching the hindbrain not only improves glycemic control (Figure 1 in Geisler et al., 2021) but decreases light cycle food intake, possibly explaining the decreased weight gain with HFD feeding in hepatic vagotomized mice (Figure 1B in Geisler et al., 2021). Further supporting a role of hepatic GABA in HFD-induced weight gain, we previously observed that inducing hepatic Kir2.1 expression limited hepatic GABA release and HFD-induced weight gain (Figure 3C in Geisler et al., 2021).

## Conclusions

Weight loss by people with obesity is often difficult to achieve and maintain. It is likely that dysregulated satiety signaling impairs the effectiveness of weight loss strategies. This work identifies hepatic GABA production as a potential therapeutic target that independently improves systemic glucose homeostasis and decreases food intake in obesity. In people with obesity, hepatic GABA production and transport are associated with glucoregulatory markers, T2D, and BMI, supporting the potential translation of this work to improve metabolic health in people.

## STAR★METHODS

### RESOURCE AVAILABILITY

#### Lead contact

Further information and requests for resources and reagents should be directed to and will be fulfilled by the lead contact, Benjamin Renquist (bjrenquist@arizona.edu).

#### Materials availability

The Antisense Oligonucleotides generated in this study are intellectual property of Ionis Pharmaceuticals (Carlsbad, CA).

#### Data and code availability

The datasets generate during this study are available at Mendeley Data: https://doi.org/10.17632/h2bww478j6.1. The RNS-seq data has been deposited into the NCBI GEO database under accession number GEO: GSE144414.

### EXPERIMENTAL MODEL AND SUBJECT DETAILS

#### Animals

All studies were conducted using male wild-type C57BL/6J mice purchased from Jackson Laboratories or bred in-house (Bar Harbor, ME). Mice were kept on a 14-hour light/10-hour dark schedule and housed 3-5 mice per cage until 1 week prior to study initiation, at which point animals were individually housed. We conducted studies in lean chow fed mice (7013 NIH-31, Teklad WI, 3.1 kcal/g, 18% kcal from fat, 59% kcal from carbohydrate, 23% kcal from protein) at 12-16 weeks of age. Studies in diet-induced obese mice dosed intraperitoneally with GABA transaminase inhibitors, mice treated with ethanolamine-O-sulfate in their drinking water, and mice treated with GABA-T targeted or scramble control antisense oligonucleotides (ASO) were performed after 8-10 weeks on a high fat diet (TD 06414, Teklad WI, 5.1 kcal/g, 60.3% kcal from fat, 21.3% kcal from carbohydrate, 18.4% kcal from protein; 20-26 weeks of age). For ASO studies, mice were stratified by body weight and assigned to an injection treatment (control or GABA-T). Studies in obese vagotomy and sham mice were performed after 9 weeks of high fat diet feeding. Unless fasted, mice had *ad libitum* access to food and water. All studies were approved by The University of Arizona Institutional Animal Care and Use Committee.

#### Hepatic Vagotomy Surgeries

Surgeries were performed in 12-week old male C57BL/6J mice under isoflurane anesthesia. Mice were randomly assigned to a surgical group (sham or vagotomy). A ventral midline incision through the skin and peritoneum allowed us to isolate the hepatic vagus nerve as it branched from the esophagus. In vagotomized mice, we severed the hepatic vagal nerve, while it remained intact in sham operated mice. The peritoneum was sutured with absorbable polyglactin 910 suture and the skin with nylon suture. Mice were given a single post-operative dose of slow release formulated buprenorphine analgesic (1.2 mg/kg slow release, sub-cutaneous). We monitored food intake and body weight daily and removed sutures 7 days post-operation.

#### Human Subjects

A total of 19 men and women with obesity (age 45 ± 3 years old, 2 men and 17 women) who were scheduled for bariatric surgery at Barnes-Jewish Hospital in St. Louis, MO participated in this study, which was conducted at Washington University School of Medicine in St. Louis. Subjects provided written, informed consent before participating in this study, which was approved by the Human Research Protection Office at Washington University School of Medicine in St. Louis, MO (ClinicalTrials.gov Database: NCT00981500).

### METHOD DETAILS

#### Antisense Oligonucleotide Studies

Wild-type lean or diet-induced obese mice received twice-weekly intraperitoneal injections (12.5 mg/kg; 0.1 mL/10 g body weight) of murine GABA-Transaminase (GABA-T) targeted antisense oligonucleotides (ASO; IONIS 1160575; 5′-AAGCTATGGACTCGGT-3′) or scramble control ASO (IONIS 549144; 5′-GGCCAATACGCCGTCA-3′) for 1 or 4 weeks prior to experimentation. The control ASO does not have complementarity to known genes and was employed to demonstrate the specificity of target reduction. 2, 4- constrained 2-O-ethyl (cEt) ASOs were synthesized at Ionis Pharmaceuticals (Carlsbad, CA) as described previously (Seth et al., 2010). Treatment with this GABA-T targeted ASO reduces hepatic GABA-T mRNA by 98% in adult mice within 1 week (Figure 2A). For studies after 1 week of ASO injections, we performed an OGTT (day 8), ITT (day 10), and mice were sacrificed to assess liver slice GABA release (day 12). For studies after 4 weeks of ASO injections, we performed an OGTT (day 29), ITT (day 30), and completed all additional studies within 1 week while continuing biweekly ASO injections before mice were sacrificed to assess liver slice GABA release.

#### GABA Transaminase Inhibitor Studies

Wild-type lean and obese mice were randomly divided into treatment groups and dosed daily with 8 mg of ethanolamine-O-sulfate (EOS; Sigma-Aldrich, St. Louis, MO), vigabatrin (United States Pharmacopeia, Rockville, MD) or PBS. Obese sham and vagotomy mice were dosed daily with 8 mg of EOS. In all cases, basal bleeds were taken prior to initiation of an ITT. Lean mice received treatment by oral gavage (0.3 mL/mouse) while obese mice were treated by intraperitoneal injection (0.3 mL/mouse). Pre-treatment studies were conducted in the week immediately prior to beginning drug administration. Daily doses took place at 9 am each day and oral glucose tolerance tests (OGTT) and insulin tolerance tests (ITT) were performed on the third and fourth days of treatment in lean mice, respectively. In wild-type obese mice, OGTT and ITT were performed on the fourth day of treatment in separate cohorts. On the fifth day of treatment we preformed 2DG clearance studies. In sham/vagotomy mice, OGTT and ITT were performed on the fourth and fifth day of treatment, respectively. After a 2-week washout period without treatment injection, a basal bleed and bleed 15 minutes after an oral glucose gavage (2.5 mg/kg) to determine oral glucose stimulated insulin secretion were performed.

In a separate set of studies, an ITT was performed in obese mice to establish insulin resistance. Subsequently, EOS was provided *ad libitum* in the water (3 g/L) for 4 days. An OGTT and ITT were performed on days 3 and 4 of treatment, respectively. The water was then removed, and an ITT was performed 2 weeks later to establish the timing of restoration of insulin resistance after drug removal.

#### Food Intake Studies

We measured food weight and body weight at 6am and 6pm for the first 14 days of control or GABA-T ASO injections in lean and obese wild-type mice (Figure 6) and GABA-T ASO injections in sham and vagotomy mice (Figure S8A–S8D). Weeks 3 and 4 food and body weight measurements were taken at 6pm on day 21 and 28 of ASO injections.

#### Fast-Induced Refeeding

Mice were housed on wood chip bedding (Harlan Laboratories; Cat #7090 Sani-Chips) to limit consumption of nutrients from bedding during the fasting period. Mice were fasted for 16 hours beginning at 5pm and food was returned at 9am. Food and body weight were measured at 10am, 11am, and 1pm to determine 1, 2, and 3-4 hour fast-induced refeeding.

#### Leptin Food Intake Study

Lean mice or diet-induced obese control or GABA-T targeted ASO treated mice received an intraperitoneal injection of phosphate buffered saline (PBS; 0.1 mL/10 g body weight) on day 1 and leptin (2 mg/kg; 0.1 mL/10 g body weight; CAT# 498-OB, R&D Systems, Minneapolis, MN) on day 2 at 6am. Mice were not fasted before injections. Food and body weight were measured every day at 6am and 6pm.

#### ASO Energy Expenditure and Body Composition

Energy expenditure studies and body composition measurements were performed by the UC Davis Mouse Metabolic Phenotyping Center. Twelve male DIO (C57BL/6J, strain 380050) mice were sent to UC Davis from the Renquist Lab at ~20 weeks of age and were acclimated for 2 weeks on investigator provided diet (Research diets D12492). Mice were injected IP with control or ABAT ASO (12.5 mg/kg) twice a week for 4 weeks (8 injections total). Energy expenditure and physical activity were evaluated in 6 saline and 6 ABAT ASO treated mice by indirect calorimetry in the CLAMS (Comprehensive Lab Animal Monitoring System, Columbus Instruments) system three times at baseline (−3), 7, and 28 days post first ASO injection. Body composition was assessed under isoflurane anesthesia by DEXA after each CLAMS run. Animals were allowed to recover from anesthesia before returning to their home cage except for the final (3^rd^) DEXA run, after which animals were terminated and no tissues collected. Animals were acclimated to the CLAMS cages for 48 hours and to the light and temperature-controlled chamber for 24 hours prior to testing. Animals were held and calorimetry data was collected for 48 hr. Analyzed data constitutes data collected from 48 hours of continuous measurement (2 light/2 dark cycles). Oxygen consumption and carbon dioxide production were measured and used to calculate energy expenditure (or heat production, kilocalories (kcal)) and respiratory exchange ratio (RER: VCO2/VO2). Cage-mounted sensors detected and recorded measurements of physical activity, food intake and water intake. Body composition was measured by dual-energy X-ray absorptiometry under isoflurane anesthesia, using a Lunar PIXImus II Densitometer (GE Medical Systems, Chalfont St. Giles, UK) immediately after completion of the indirect respiration calorimetry measurements.

#### EOS Body Composition

Body composition was assessed in diet-induced obese mice on day 0 and 7 days after continuous provision of EOS in the drinking water (3 g/L) using an EchoMRI 900 with A10 insert for mice. We performed calibration daily and had the water stage set to on.

#### Hyperinsulinemic Euglycemic Clamps

Clamps were performed as previously described (Ayala et al., 2011). Briefly, one week prior to the experiment, diet-induced obese mice underwent surgical catheterization of the jugular vein under isoflurane anesthesia. Mice were given a single post-operative dose of slow release formulated buprenorphine analgesic (1.2 mg/kg slow release, sub-cutaneous) and food and body weight were assessed daily for 1 week. The same day following surgery completion mice received their first injection of either the scramble control or GABA-T targeted ASO (12.5 mg/kg; 0.1 mL/10 g body weight). Four days post-surgery mice received a second ASO injection, and clamps were performed 7 days after catheterization. Following a 5-h fast (starting at 0800h), clamps were performed using unrestrained, conscious mice. The clamp procedure consisted of a 90-min tracer equilibration period (−90 to 0 min), followed by a 120-min clamp period (0 to 120 min). To begin the equilibration period, mice were infused with 3-[^3^H]-D-glucose (0.05 μCi/μl in saline) at a rate of 10 μL/min for 2 minutes and then decreased to a rate of 1 μL/min for the remaining 90 minutes. All blood was collected from the tip of the tail in heparinized capillary tubes and immediately spun down to collect plasma for tracer and insulin analysis. All time points during the clamp are relative to the start of insulin infusion at time 0. Blood for tracer and insulin analysis was taken at −10 and 0 minutes. At 0 minutes mice were infused with donor blood (5 μL/min continuously throughout study), insulin (4 mU/kg/min; pump rate 1 μL/min continuously throughout study), and a variable infusion of 3-[^3^H]-D-glucose (0.05 μCi/μl in 50% dextrose) to maintain euglycemia. Glucose was assessed from whole-blood directly from the tail tip by glucometer (Manufacture # D2ASCCONKIT, Bayer, Leverkusen, Germany) every 10 minutes for 120 minutes starting at time 0. The glucose infusion rate was adjusted to maintain a blood glucose concentration in the range of 100-120 mg/dL. Blood for tracer measurements was taken at 80, 90, 100, and 120 minutes, and blood for insulin was taken at 100 and 120 minutes. To estimate insulin-stimulated glucose fluxes in tissues, mice were given a bolus of ^14^C-2-deoxyglucose (12 μCi in 48 μL followed by a 100 μL saline flush) after the 120min time point. Blood samples were collected 2, 10, and 25 minutes following the bolus. Mice were anesthetized with isoflurane using the bell-jar method and the soleus, quadricep, calf, white adipose tissue, and perirenal adipose tissue were collected and immediately frozen in liquid nitrogen for analysis of tissue specific glucose uptake. Plasma samples were deproteinized using barium hydroxide and zinc sulfate and tracer analysis and tracer standards was processed as described (Ayala et al., 2011). Plasma glucose was analyzed by colorimetric assay (Cat. # G7519, Pointe Scientific Inc., Canton MI) from the samples collected for tracer measurement. Plasma insulin was analyzed by enzyme-linked immunosorbent assay (ELISA; Cat. # 80-INSMSU-E01,E10, Alpco, Salem, NH).

#### Oral Glucose Tolerance Test

Oral glucose (2.5g/kg; 0.1 mL/10 g body weight; Chem-Impex Int’l Inc., Wood Dale, IL) was given to 4 hour fasted individually housed mice. All glucose tolerance tests began at 1 pm and glucose was measured in whole blood, collected from the tail vein, by glucometer (Manufacture # D2ASCCONKIT, Bayer, Leverkusen, Germany) at 0, 15, 30, 60, 90, and 120 minutes after glucose gavage. Blood for serum insulin (oral glucose stimulated insulin secretion; OGSIS) and glucose determination was collected from the tail vein 15 minutes following glucose administration.

#### Insulin Tolerance Test

Intraperitoneal insulin (0.5 U/kg; 0.1 mL/10 g body weight; Sigma Aldrich, St. Louis, MO) was given to 4 hour fasted individually housed mice. All insulin tolerance tests began at 1 pm and glucose was measured in whole blood, collected from the tail vein, by glucometer (Manufacture # D2ASCCONKIT, Bayer, Leverkusen, Germany) at 0, 30, 60, 90, and 120 minutes after insulin injection.

#### Liver Slice Explant Studies

Liver slices from ASO treated mice were incubated *ex vivo* to measure hepatic GABA release. A peristaltic pump perfusion system was used to deliver warmed KH buffer to the liver through the portal vein. Briefly, mice were anesthetized with an intraperitoneal injection of ketamine (10 mg/mL) and diazepam (0.5 mg/mL). Once mice were unresponsive, an incision in the lower abdomen through the skin and peritoneal membrane was made vertically through the chest along with transverse incisions on both sides to expose the liver. A 30-gauge needle was inserted into the hepatoportal vein to blanch the liver. The inferior vena cava was cut to relieve pressure in the circulatory system and allow blood to drain. The perfusion continued for several minutes at a rate of 4 mL/minute until the liver was completely blanched. The liver was removed and washed in warm PBS before being sliced into 0.2 mm slices using a Thomas Sadie-Riggs Tissue Slicer. Two liver slices were taken from each mouse. Tissue slices were placed individually into a well on a 12-well plate pre-filled with 1mL of KH buffer that had been sitting in an incubator set to 37°C and gassed with 5% CO_2_. Liver slices were incubated in the initial well for 1 hour to stabilize before being transferred to a fresh well pre-filled with KH buffer. After 1 hour in the second well, tissue and media were collected. Liver slice samples and KH media samples from both wells of each mouse were pooled. Liver slices were snap frozen in liquid nitrogen, while media was centrifuged for 5 minutes at 10,000xg at 4°C to remove tissue debris and both were frozen and stored at −80°C pending analysis.

#### GABA Analysis

For all liver slice GABA release data, we thawed the media collected from the *ex vivo* hepatic slice culture on ice. We then measured GABA in the supernatant using a commercially available ELISA (REF# BA E-2500, Labor Diagnostika Nord, Nordhorn, Germany). μmol GABA concentrations were corrected for liver slice DNA concentrations.

#### ^3^H-2-deoxy-D-glucose Uptake Studies

On the fifth day of EOS or PBS treatment ^3^H-2-deoxy-D-glucose (2DG; 10 uCi/mouse; PerkinElmer, Waltham, MA) was given to 4-hour fasted individually housed mice. Studies were performed in 2 cohorts on 2 different days. 2DG was given by oral gavage in a solution of glucose (2.5 g/kg) and each mouse received the same dose based off the average body weight for their cohort (0.1 mL/10 g body weight). Mice were anesthetized by isoflurane and sacrificed by cervical dislocation 45 minutes following oral gavage. Liver, soleus, quadriceps femoris, and gonadal white adipose tissue were collected, weighed, and dissolved overnight in 1N NaOH (0.5 mL/50 mg tissue) at 55°C on a shaker plate. 0.5 mL of dissolved tissue was added to 5 mL of scintillation cocktail (Ultima Gold, PerkinElmer, Waltham, MA) and disintegrations per minute (DPM) were measured using a LS 6500 Multipurpose Scintillation Counter (Beckman Coulter, Brea, CA). DPM/g tissue weight was determined for each tissue and normalized based on a correction factor calculated by the average total DPM/g for all tissues divided by the total DPM/g for all tissues of the individual mouse.

#### cGMP Analysis

On the fifth day of EOS or PBS treatment, mice were sacrificed and the quadricep and soleus tissues were collected and frozen on dry ice. Prior to analysis, frozen quadricep were powdered using a liquid nitrogen cooled mortar and pestle to obtain homogeneous muscle samples. 15-20 mg of quadricep and the entire soleus tissue (6-12 mg) were homogenized in 200 μL of a 5% trichloroacetic acid solution. Following 15 minutes of centrifugation at 3,000xg at 4°C, supernatant was transferred to a fresh tube for analysis of muscle cGMP by enzyme-linked immunosorbent assay (ELISA; ADI-900-164, Enzo Life Sciences, Farmingdale, NY).

#### Liver Analysis

Prior to analysis, frozen livers were powdered using a liquid nitrogen cooled mortar and pestle to obtain homogeneous liver samples. To measure liver DNA content (ng dsDNA/g tissue), 10-20 mg of powdered liver was sonicated in 500 μL DEPC H_2_O and dsDNA determined by fluorometric assay (Cat. # P7589, Invitrogen, Waltham, MA). Whole liver and hypothalamic mRNA were isolated from powered liver samples with TRI Reagent® (Life Technologies, Grand Island, NY) and phenol contamination was eliminated by using water-saturated butanol and ether as previously described (Krebs et al., 2009). cDNA was synthesized by reverse transcription with Verso cDNA synthesis kit (Thermo Scientific, Inc., Waltham, MA), and qPCR performed using SYBR 2X mastermix (Bio-Rad Laboratories, Hercules, CA) and the Biorad iQ™5 iCycler (Bio-Rad Laboratories, Hercules, CA). Expression of GABA-Transaminase (ABAT), β-actin (ACTβ), insulin (Ins), neuropeptide Y (NPY), agouti related peptide (AgRP), and pro-opiomelanocortin (POMC) mRNA were measured using the following primers (5′→3′): ABAT, Forward – CTGAACACAATCCAGAATGCAGA Reverse – GGTTGTAACCTATGGGCACAG; ACTβ, Forward – TCGGTGACATCAAAGAGAAG Reverse – GATGCCACAGGATTCCATA; Ins, Forward – GCTTCTTCTACACACCCATGTC Reverse – AGCACTGATCTACAATGCCAC; NPY, Forward – CTCGTGTGTTTGGGCATTCT Reverse – CTTGCCATATCTCTGTCTGGTG; AgRP, Forward – GTACGGCCACGAACCTCTGT Reverse – TCCCCTGCCTTTGCCCAA; POMC, Forward – GGTGAAGGTGTACCCCAACGT Reverse – GACCTGGCTCCAAGCCTAATGG. LinReg PCR analysis software was used to determine the efficiency of amplification from raw output data (Ramakers et al., 2003). ACTβ served as the reference gene for liver and brain tissue, and Ins served as the reference gene for pancreas tissue for calculating fold change in ABAT gene expression using the efficiency^−ΔΔCt^ method (Livak and Schmittgen, 2001).

#### Serum Assays

Within 2 hours of collection, blood was left to clot at room temperature for 20 minutes. Thereafter, the blood was centrifuged at 3,000xg for 30 minutes at 4°C and serum was collected. Serum was stored at −80°C until metabolite and hormone analyses. We used a colorimetric assay to analyze serum glucose (Cat. # G7519, Pointe Scientific Inc., Canton MI). Serum insulin was analyzed by enzyme-linked immunosorbent assay (ELISA; Cat. # 80-INSMSU-E01,E10, Alpco, Salem, NH). Serum glucagon was analyzed by enzyme-linked immunosorbent assay (ELISA; Cat. # 10-1281-01, Mercodia, Uppsala, Sweden) from tail vein blood collected at 9 am from fed mice.

#### Studies Conducted in Human Subjects

Intrahepatic triglyceride content was determined by using magnetic resonance imaging (3.0-T superconducting magnet; Siemens, Iselin, NJ) in the Center for Clinical Imaging Research. A 7-hour (3.5-h basal period and 3.5-h insulin infusion period) hyperinsulinemic-euglycemic clamp procedure (HECP), in conjunction with stable isotopically labeled glucose tracer infusion, was then conducted in the Clinical Translational Research Unit (CTRU), as previously described (Korenblat et al., 2008). This procedure was performed to determine: i) hepatic insulin sensitivity, which was assessed as the product of the basal endogenous glucose production rate (in μmol·kg fat-free mass (FFM)^−1^ · min^−1^) and fasting plasma insulin concentration (in mU/L). For liver RNA sequencing (RNA-seq), liver tissue was obtained by needle biopsy during the bariatric surgical procedure, before any intra-operative procedures were performed. Liver tissue was rinsed in sterile saline, immediately frozen in liquid nitrogen, then stored at −80°C until RNA extraction. Total RNA was isolated from frozen hepatic tissue samples by using Trizol reagent (Invitrogen, Carlsbad, CA). Library preparation was performed with total RNA and cDNA fragments were sequenced on an Illumina HiSeq-4000. The fragments per kilobase million reads upper quartile (FPKM-UQ) values were calculated and used for further gene expression analyses. All RNA-seq data used in this study have been deposited into the NCBI GEO database under accession number GEO: GSE144414.

### QUANTIFICATION AND STATISTICAL ANALYSIS

#### Statistics

We analyzed the data in SAS Enterprise Guide 7.1 (SAS Inst., Cary, NC), using a mixed-model ANOVA for all analyses except the multivariate regression analyses performed on human clinical data. ANOVA tests do not have a one-tailed versus two-tailed option, because the distributions they are based on have only one tail. When comparisons between all means were required, we used a Tukey’s adjustment for multiple comparisons. When comparisons of means were limited (e.g., only within a time point or treatment), we used a Bonferonni correction for multiple comparisons. For the analysis of ITT and OGTT, repeated-measures ANOVA were performed by including time point in the analysis. Analyses were conducted separately for chow and HFD fed mice. For the studies using the GABA-T inhibitors the main effect was treatment (PBS, Vigabatrin, or EOS). For EOS treated sham and vagotomy mice the main effects were surgery (sham or vagotomy) and treatment (PBS or EOS). Pre-, during, and post-treatment measures were taken for basal glucose, insulin, and glucose stimulated insulin, thus a repeated-measure analysis including time (pre-, during, or post-treatment) was performed separately within each injection or surgical treatment. For analysis of the effect of EOS on 2DG uptake and cGMP, analysis was performed separately for each tissue. For ASO studies the main effect was injection treatment (control of GABA-T ASO). For the vagotomy analyses the main effect was surgery (sham or vagotomy). Pre-treatment and ASO week 4 measures were taken for basal glucose, insulin, and the glucose:insulin ratio, thus a repeated-measure analysis including time (pre-, or week 4-treatment) was performed separately within each injection or surgical treatment. A multivariate regression analysis was performed on data from human clinical patients using IHTG content, *ABAT* mRNA, and *SLC6a12* mRNA as explanatory variables for variations in serum insulin, HOMA-IR, M-Value, and Glucose Rd. Statistics performed by the UC Davis Mouse Metabolic Phenotyping Center are described as follows: data are presented as means ± SEM and a Student t test was used to test for significant differences between groups. Multiple linear regression analysis (analysis of covariance, ANCOVA) was used to assess the impact of covariates (e.g., body weight or lean mass) on energy expenditure. The EE ANCOVA analysis done for this work was provided by the NIDDK Mouse Metabolic Phenotyping Centers (MMPC, https://www.mmpc.org) using their Energy Expenditure Analysis page (https://www.mmpc.org/shared/regression.aspx) and supported by grant DK076169. All insulin tolerance tests are presented as a percentage of baseline glucose in main and supplemental figures, and additionally presented as raw glucose values in Figure S9. All graphs were generated using GraphPad Prism 8 (GraphPad Software Inc., La Jolla, CA). Information on replicates and significance is included in the figures. Error bars are defined in the figure legends.

## Supplementary Material

1

## Figures and Tables

**Table:** KEY RESOURCES TABLE

REAGENT or RESOURCE	SOURCE	IDENTIFIER
Chemicals, peptides, and recombinant proteins		
D-glucose	Chem-Impex Int’l Inc.	Cat # 00805
Insulin	Sigma Aldrich	Cat# I0516-5ML
Leptin	R&D Systems	Cat # 498-OB
Ethanolamine-O-sulfate (EOS)	Sigma Aldrich	Cat # 06720-100G
Vigabatrin	United States Pharmacopeia	Cat # 1712001
H^3^-2-deoxy-D-glucose	Perkin Elmer	Cat # NET328A001MC
3-[^3^H]-D-glucose	Perkin Elmer	Cat# NET331C001MC
^14^C-2-deoxyglucose	Perkin Elmer	Cat# NEC495A001MC
TRIzol® LS Reagent	Life Technologies	Cat # 10296010
iTaq™ Universal SYBR® Green Supermix	Bio-Rad Laboratories	Cat # 172-5121
Critical commercial assays		
Glucose Oxidase Assay	Pointe Scientific Inc.	Cat # G7519
Insulin ELISA	Alpco	Cat # 80-INSMSU-E01,E10
GABA ELISA	Labor Diagnostika Nord	Cat # BA E-2500
Glucagon ELISA	Mercodia	Cat # 10-1281-01
cGMP ELISA	Enzo Life Sciences	Cat # ADI-900-164
Quant-iT PicoGreen dsDNA Assay Kit	Invitrogen	Cat # P7589
Verso cDNA Synthesis Kit	Thermo Scientific Inc.	Cat # AB-1453/B
Deposited data		
Raw Data	This Paper	Mendeley Data: https://doi.org/10.17632/h2bww478j6.1
RNA-seq Data	This Paper	NCBI GEO: GSE144414
Experimental models: organisms/strains		
Mouse: C57BL6/J	Jackson Laboratories	JAX stock 000664
Oligonucleotides		
5′→3′ACTβ F: TCGGTGACATCAAAGAGAAG	Integrated DNA Technologies	N/A
5′→3′ACTβ R: GATGCCACAGGATTCCATA	Integrated DNA Technologies	N/A
5′→3′ABAT F: CTGAACACAATCCAGAATGCAGA	Integrated DNA Technologies	N/A
5′→3′ABAT R: GGTTGTAACCTATGGGCACAG	Integrated DNA Technologies	N/A
5′→3′ INS F: GCTTCTTCTACACACCCATGTC	Integrated DNA Technologies	N/A
5′→3′ INS R: AGCACTGATCTACAATGCCAC	Integrated DNA Technologies	N/A
5′→3′ NPY F: CTCGTGTGTTTGGGCATTCT	Integrated DNA Technologies	N/A
5′→3′ NPY R: CTTGCCATATCTCTGTCTGGTG	Integrated DNA Technologies	N/A
5′→3′ AgRP F: GTACGGCCACGAACCTCTGT	Integrated DNA Technologies	N/A
5′→3′ AgRP R: TCCCCTGCCTTTGCCCAA	Integrated DNA Technologies	N/A
5′→3′ POMC F: GGTGAAGGTGTACCCCAACGT	Integrated DNA Technologies	N/A
5′→3′ POMC R: GACCTGGCTCCAAGCCTAATGG.	Integrated DNA Technologies	N/A
5′→3′GABA-T ASO: AAGCTATGGACTCGGT	IONIS Pharmaceuticals, Inc.	Cat # 1160575
5′→3′Scramble control ASO: GGCCAATACGCCGTCA	IONIS Pharmaceuticals, Inc.	Cat # 549144
Software and algorithms		
SAS Enterprise Guide 7.1	SAS Institute Inc.	https://www.sas.com/en_us/home.html
GraphPad Prism 8	GraphPad Software Inc.	https://www.graphpad.com/
LinReg PCR Analysis Software	Heart Failure Research Center	http://linregpcr.nl
Other		
Mouse Chow Food	Teklad	7013 NIH-31
Mouse 60% High Fat Diet Food	Teklad	TD 06414

## Highlights

- Liver GABA-T knockdown or inhibition decreases serum insulin in DIO mice
- Liver GABA-T knockdown improves insulin sensitivity and muscle glucose uptake
- Liver GABA-T knockdown decreases food intake and body mass in DIO mice
- Liver GABA-T expression is positively correlated with insulin in people with obesity
