# Supplementary material for: A critical role of hepatic GABA in the metabolic dysfunction and hyperphagia of obesity
Source: Cell Rep. Author manuscript; Available in PMC 2022 Feb 17. (PMC8851954; doi:10.1016/j.celrep.2021.109301)
Supplement: 1 [file NIHMS1777623-supplement-1.pdf]

Cell Reports, Volume *35*

## Supplemental information

# A critical role of hepatic GABA in the metabolic dysfunction and hyperphagia of obesity

**Caroline E. Geisler, Susma Ghimire, Stephanie M. Bruggink, Kendra E. Miller, Savanna N. Weninger, Jason M. Kronenfeld, Jun Yoshino, Samuel Klein, Frank A. Duca, and Benjamin J. Renquist**

**Supplemental Titles and Legends**

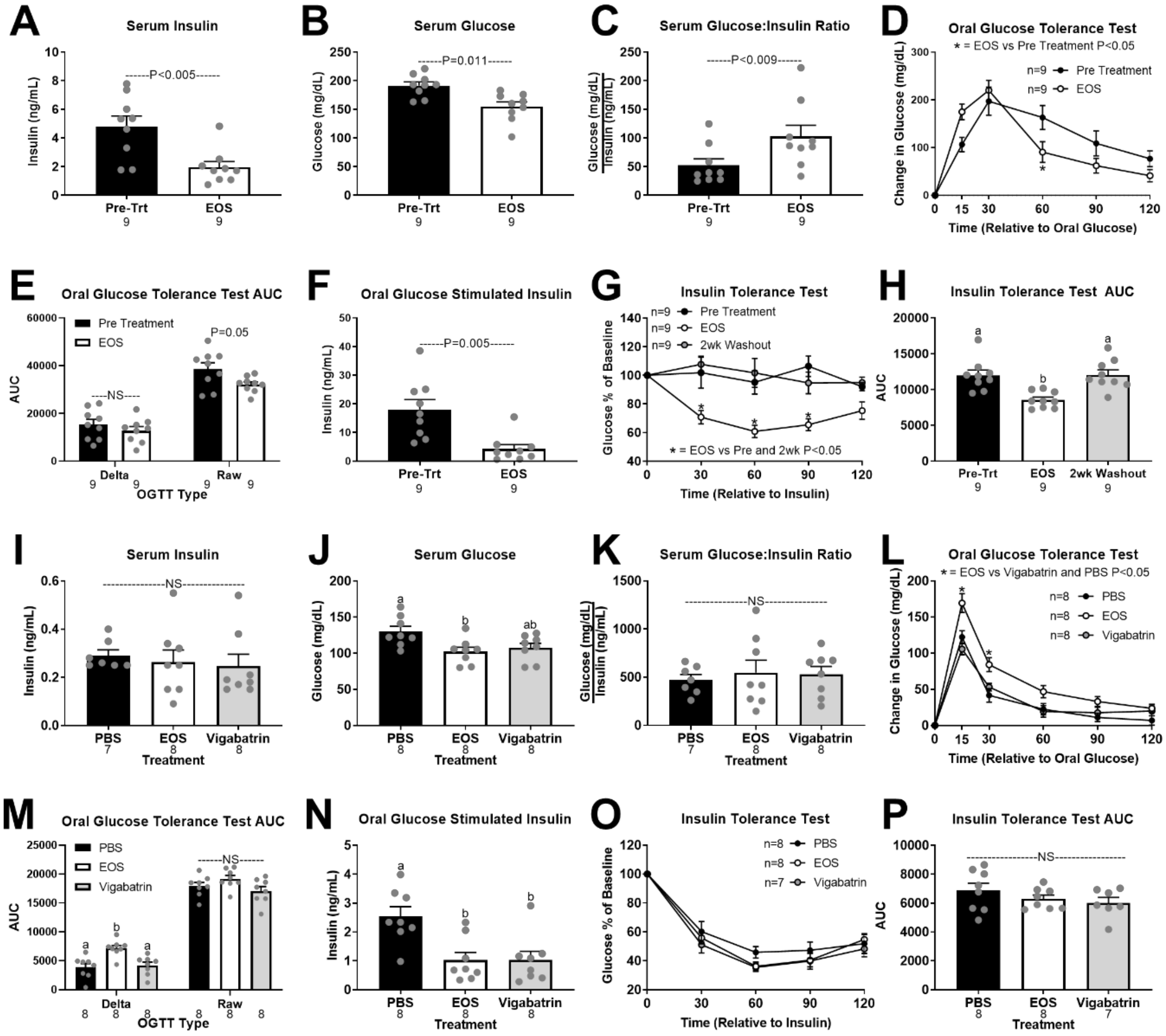


**Figure S1.** Related to Figure 1. Effects of GABA-Transaminase inhibition in obese (A-H) and lean (I-P) male mice. (A-H) Glucose homeostasis in obese male mice treated with the GABA-Transaminase inhibitor ethanolamine-O-sulfate (EOS; 3 g/L in drinking water). EOS effects on serum insulin (A) glucose (B), and glucose:insulin ratio (C) pre-treatment and after 4 days of treatment. Oral glucose tolerance (OGTT; D) OGTT area under the curve (OGTT AUC; E), and oral glucose stimulated insulin (F) pre-treatment and after 3 days of treatment. Insulin tolerance (ITT; G) and ITT AUC (H) pre-treatment, on day 4 of treatment (EOS), and after a 2-week washout period. (I-P) Glucose homeostasis in lean male mice treated with GABA-Transaminase inhibitors EOS or vigabatrin (8 mg/day), or phosphate buffered saline (PBS; control). Serum insulin (I), glucose (J), and glucose:insulin ratio (K) on treatment day 4. Oral glucose tolerance (OGTT; L), OGTT AUC (M), and oral glucose stimulated serum insulin (N) on treatment day 3. Insulin tolerance (ITT; O) and ITT AUC (P) on treatment day 4. NS = non-significant. [a,b] Bars that do not share a common letter differ significantly ($P < 0.05$; number below bar denotes n per group). All data are presented as mean ± SEM.

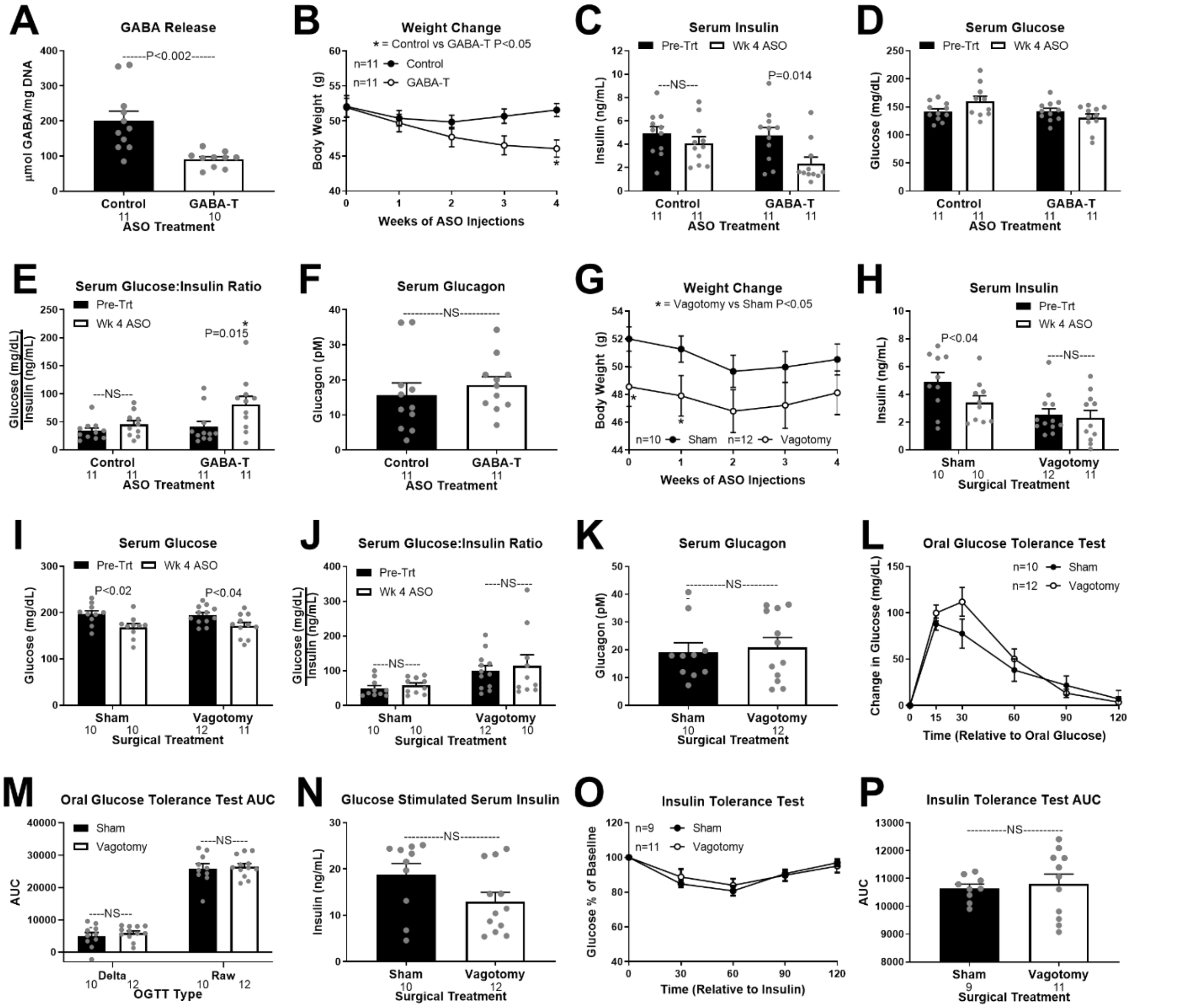


**Figure S2.** Related to Figure 2. Chronic hepatic GABA-Transaminase knockdown improves obesity induced metabolic dysfunction (A-F) and is dependent on an intact hepatic vagal nerve (G-P). (A-F) High fat diet-induced obese mice were treated for 4 weeks with a GABA-T targeted or scramble control antisense oligonucleotide (ASO; 12.5 mg/kg IP twice weekly). Release of GABA (μmol/mg DNA) from hepatic slices (A). Body weight during treatment (B). Basal serum insulin (C), glucose (D), and glucose:insulin ratio (E) pre-treatment and after 4 weeks of treatment. Serum glucagon (F) after 4 weeks of treatment. (G-P) Diet-induced obese hepatic vagotomized and sham operated mice were treated with a GABA-T targeted ASO (12.5 mg/kg IP twice weekly) for 4 weeks. Body weight during treatment (G). Basal serum insulin (H), glucose (I), and glucose:insulin ratio (J) pre-treatment and after 4 weeks of treatment. Serum glucagon (K), oral glucose tolerance (OGTT; L), OGTT area under the curve (AUC; M), oral glucose stimulated serum insulin (N), insulin tolerance (ITT; O), and ITT AUC (P) after 4 weeks of treatment. Number below bar denotes n per group. NS = non-significant. All data are presented as mean ± SEM.

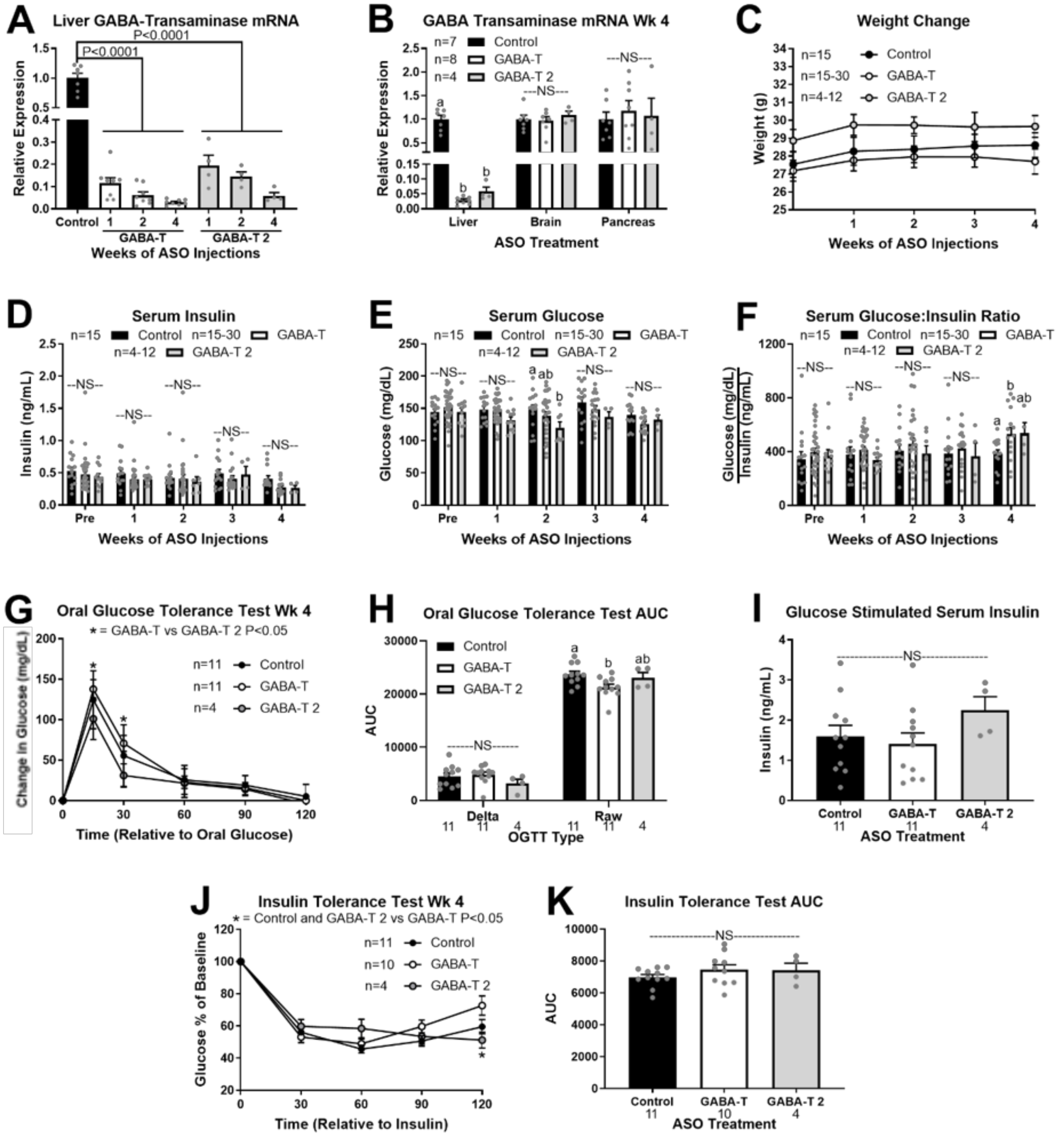


**Figure S3.** Related to Figure 2. Glucose homeostasis in lean mice treated with the scramble control antisense oligonucleotide (ASO), or 1 of 2 GABA-Transaminase (GABA-T) targeted ASO sequences (GABA-T or GABA-T 2; 12.5 mg/kg IP twice weekly) for 4 weeks. Hepatic GABA-T mRNA expression after 1, 2, and 4 weeks of ASO injections (A). GABA-T mRNA expression in liver, whole-brain, and pancreas after 4 weeks of ASO injections (B). Body weight during treatment (C). Basal serum insulin (D), glucose (E), and glucose:insulin ratio (F) pre-treatment and after 1, 2, 3, and 4 weeks of treatment. Oral glucose tolerance (OGTT; G), OGTT area under the curve (AUC; H), oral glucose stimulated serum insulin (I), insulin tolerance (ITT; J), and ITT AUC (K). [a,b,c] Bars that do not share a common letter differ significantly ($P < 0.05$). Number below bar denotes n per group. NS = non-significant. All data are presented as mean ± SEM.

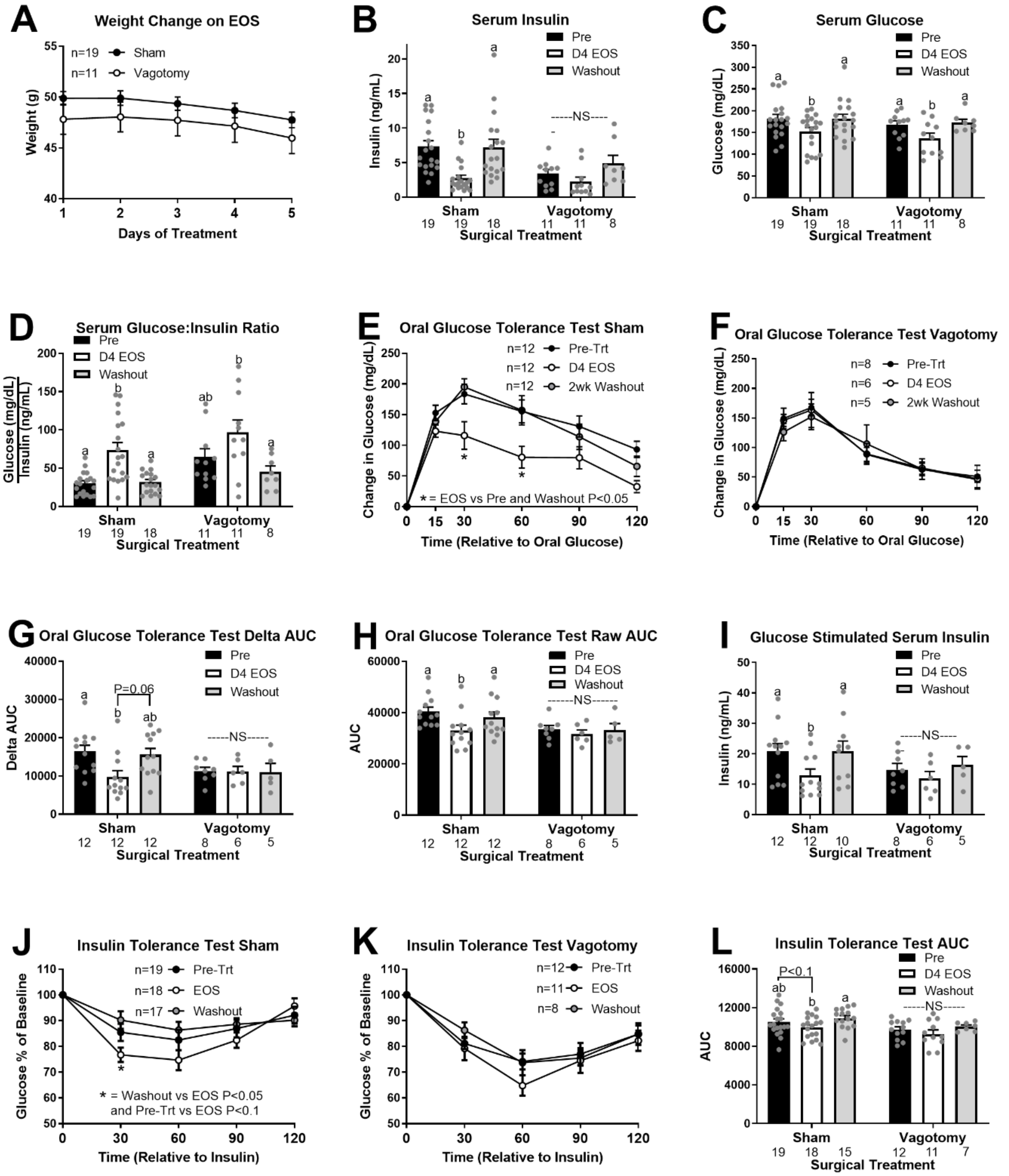


**Figure S4.** Related to Figure 1. GABA-Transaminase inhibition improves glucose homeostasis in sham but not vagotomy mice. HFD induced sham operated and hepatic vagotomized mice were treated with the GABA-Transaminase inhibitor ethanolamine-O-sulfate (EOS) (8mg/day) for 5 days. Body weight during treatment (A). Basal serum insulin (B), glucose (C), and glucose:insulin ratio (D) pre-treatment, on treatment day 5, and after a 2-week washout. Oral glucose tolerance in sham mice (OGTT; E), oral glucose tolerance in vagotomized mice (F) OGTT area under the curve (AUC; G), and glucose stimulated serum insulin (H) pre-treatment, on treatment day 4, and after a 2-week washout. Insulin tolerance in sham mice (ITT; I) and vagotomized mice (J), and ITT AUC (K) at pre-treatment, on treatment day 5, and after a 2-week washout. NS = non-significant. [a,b] Bars that do not share a common letter differ significantly within

injection treatment ($P < 0.05$; number below bar denotes n per group). All data are presented as mean ± SEM.

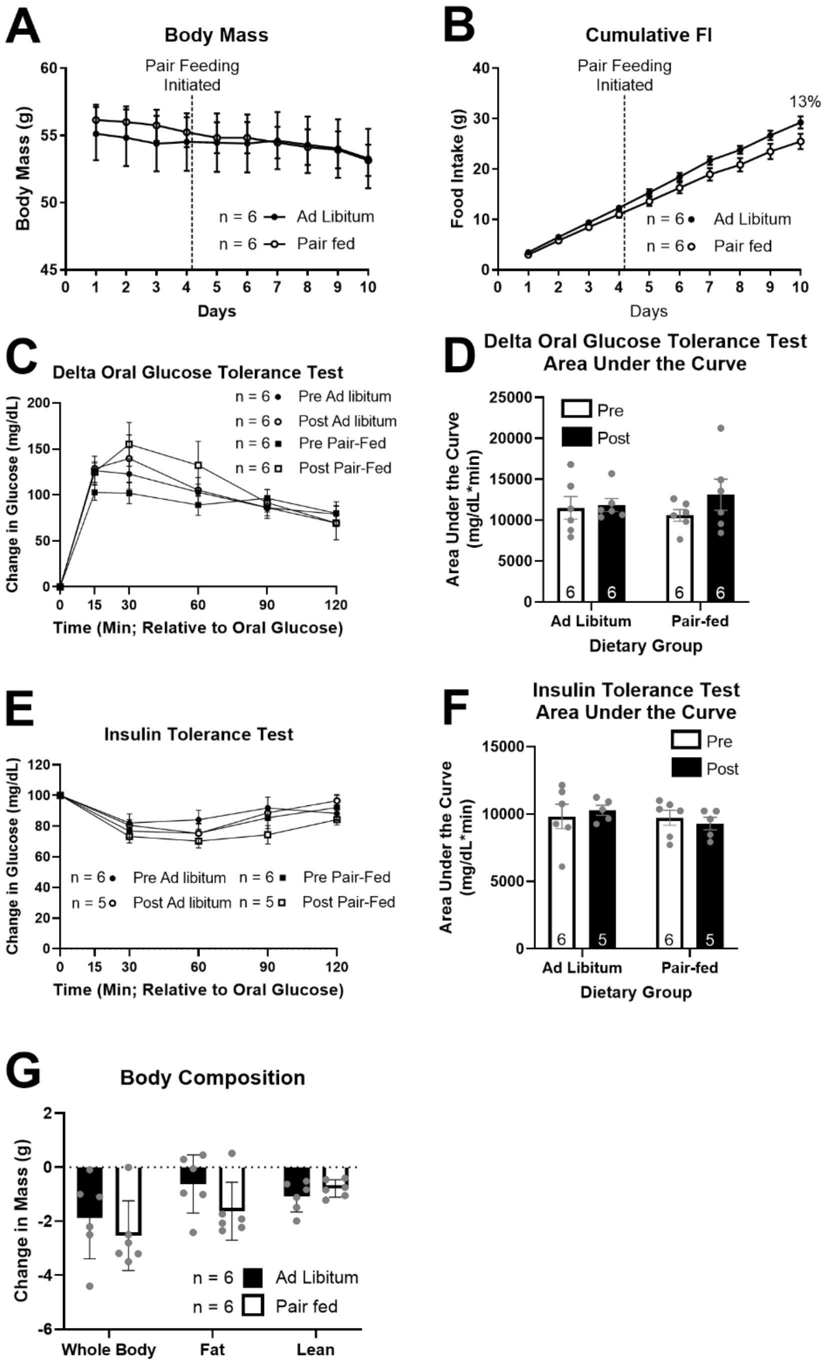


**Figure S5.** Related to Figure 2. Pair-feeding to equal the caloric restriction that results from GABA-T knockdown does not explain the metabolic improvements associated with GABA-T ASO treatment. Pair feeding did not affect body mass (A), decreased cumulative food intake (FI) by 13% relative to *ad libitum* fed controls (B), and had no effect of change in glucose during an oral glucose tolerance test (C and D), insulin sensitivity (E and F), or body mass, fat mass or lean mass loss (G). Number below bar denotes n per group. NS = non-significant. All data are presented as mean ± SEM.

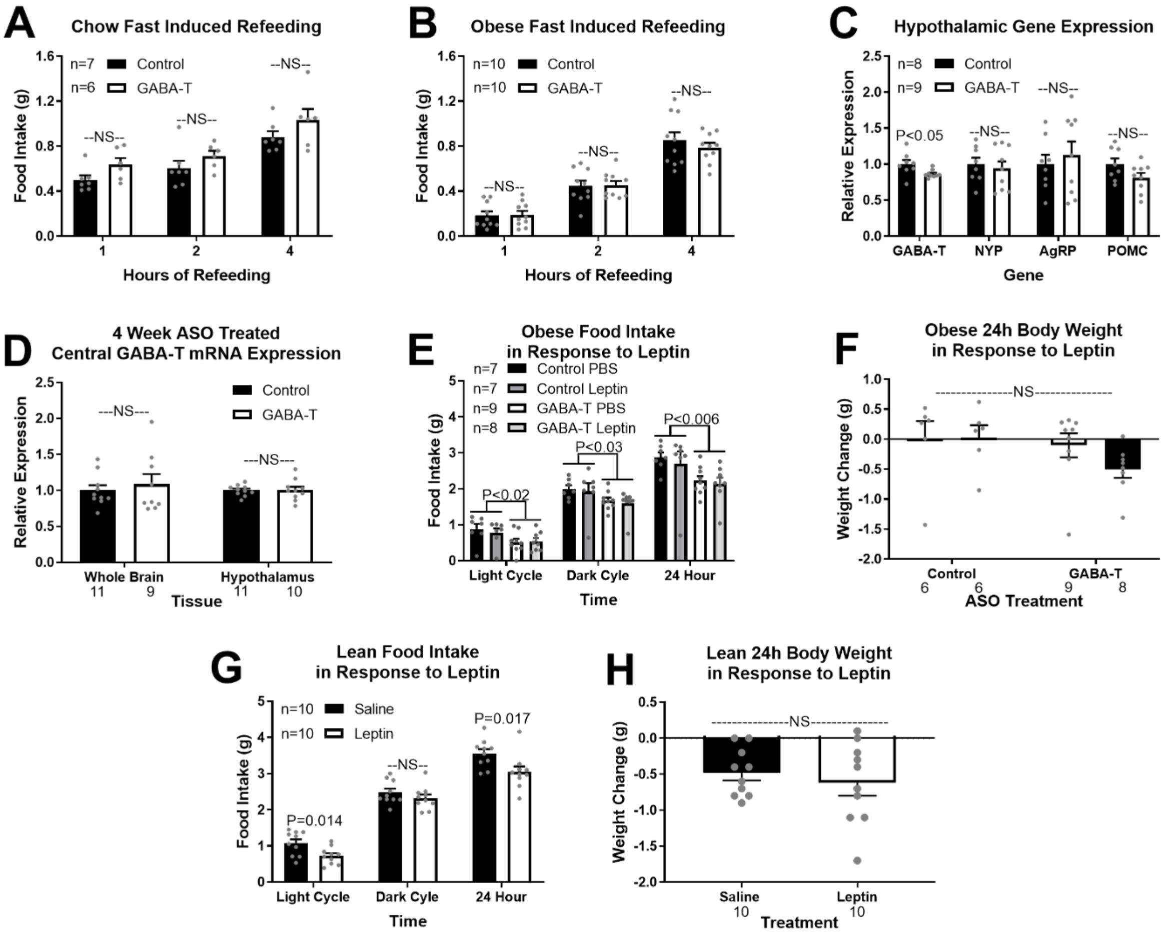


**Figure S6.** Related to Figure 4. Hepatic GABA-Transaminase knockdown does not affect fast induced refeeding or leptin sensitivity. Refeeding after a 16-hour fast in chow fed lean (A) and diet induced obese (B) mice after 4 weeks of GABA-T targeted or scramble control ASO injections (12.5 mg/kg IP twice weekly). Hypothalamic fasted mRNA expression of GABA-T, neuropeptide Y (NPY), agouti related peptide (AgRP), and pro-opiomelanocortin (POMC) after 7 weeks of ASO injections. Central GABA-T mRNA expression after 4 weeks of ASO injections (D). The effect of leptin (2 mg/kg IP single injection at 6am) on food intake (E) and body weight change (F) in obese control and GABA-T knockdown mice, and food intake (G) and body weight change (H) in lean mice. Number below bar denotes n per group. NS = non-significant. All data are presented as mean ± SEM.

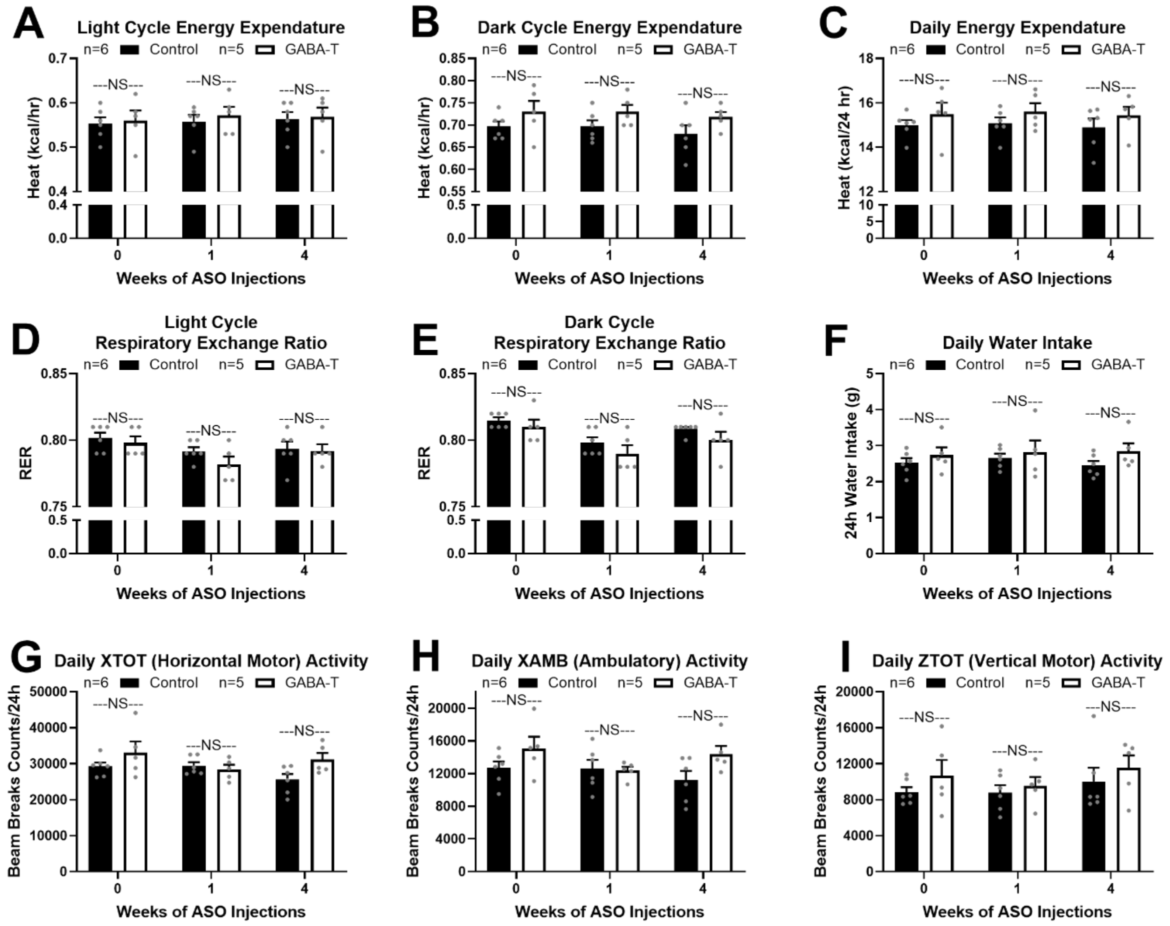


**Figure S7.** Related to Figure 5. Hepatic GABA-T knockdown does not alter energy expenditure in obesity. Energy expenditure, respiratory exchange ratio, and activity level were assessed by Comprehensive Lab Animal Monitoring System (CLAMS) at the UCDavis Mouse Metabolic Phenotyping Center in diet-induced obese mice after 0, 1, and 4 weeks of GABA-T targeted or scramble control antisense oligonucleotide treatment (ASO; 12.5 mg/kg IP twice weekly). Energy expenditure during the light cycle (A), dark cycle (B), and over 24 hours (C). Respiratory exchange ratio (RER) during the light cycle (D) and dark cycle (E). 24 hour water intake (F). 24 hour activity along the horizontal X axis (XTOT; G), total ambulatory movement (XAMB; H), and vertical Z axis (ZTOT; I). NS = non-significant. All data are presented as mean ± SEM.

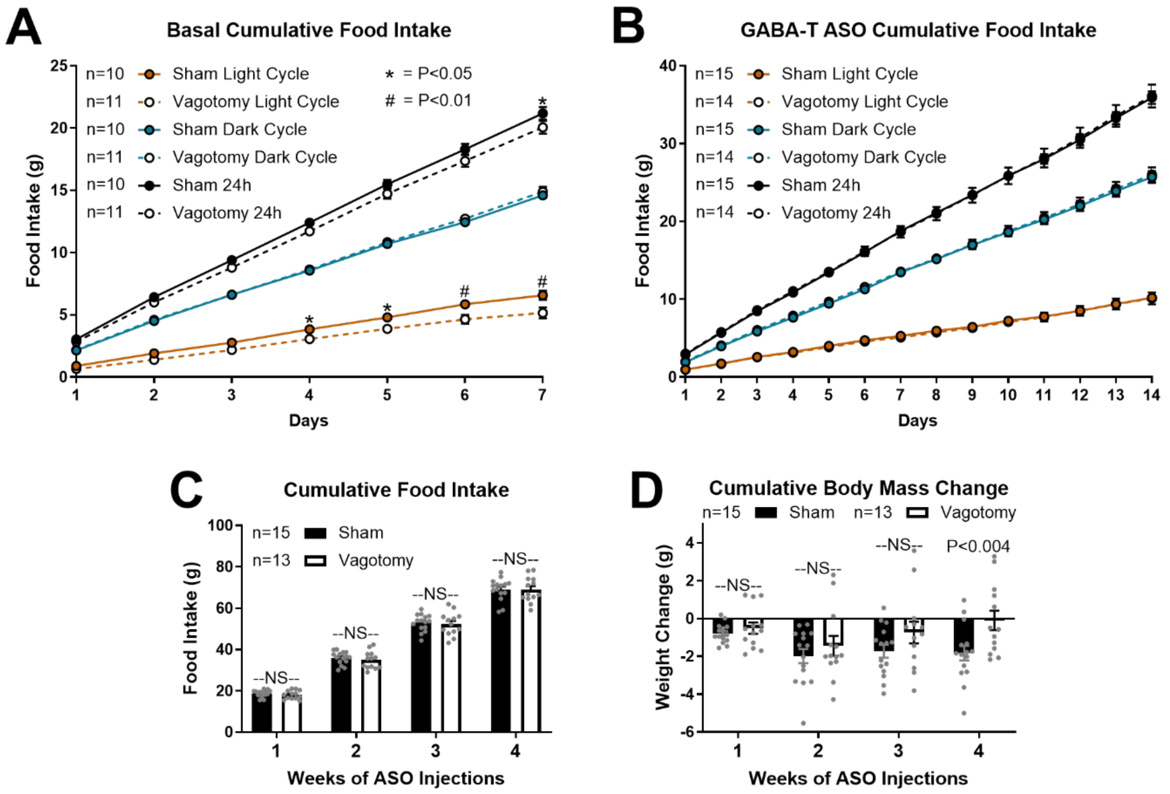


**Figure S8.** Related to Figure 4. Hepatic vagotomy decreases light cycle food intake on HFD, while GABA-Transaminase knockdown normalizes sham mice food intake to vagotomy mice. Cumulative food intake and body weight in diet-induced obese sham operated and hepatic vagotomized mice during 1 week of baseline feeding (A-B) and during 2 weeks of GABA-T targeted antisense oligonucleotide injections (ASO; 12.5 mg/kg IP twice weekly; C-F). Cumulative basal light cycle, dark cycle, and daily food intake (A) and cumulative body weight change (B). Cumulative ASO light cycle, dark cycle, and daily food intake (C) and cumulative body weight change (D). Weekly cumulative food intake (E) and cumulative body weight change (F). All data are presented as mean ± SEM.

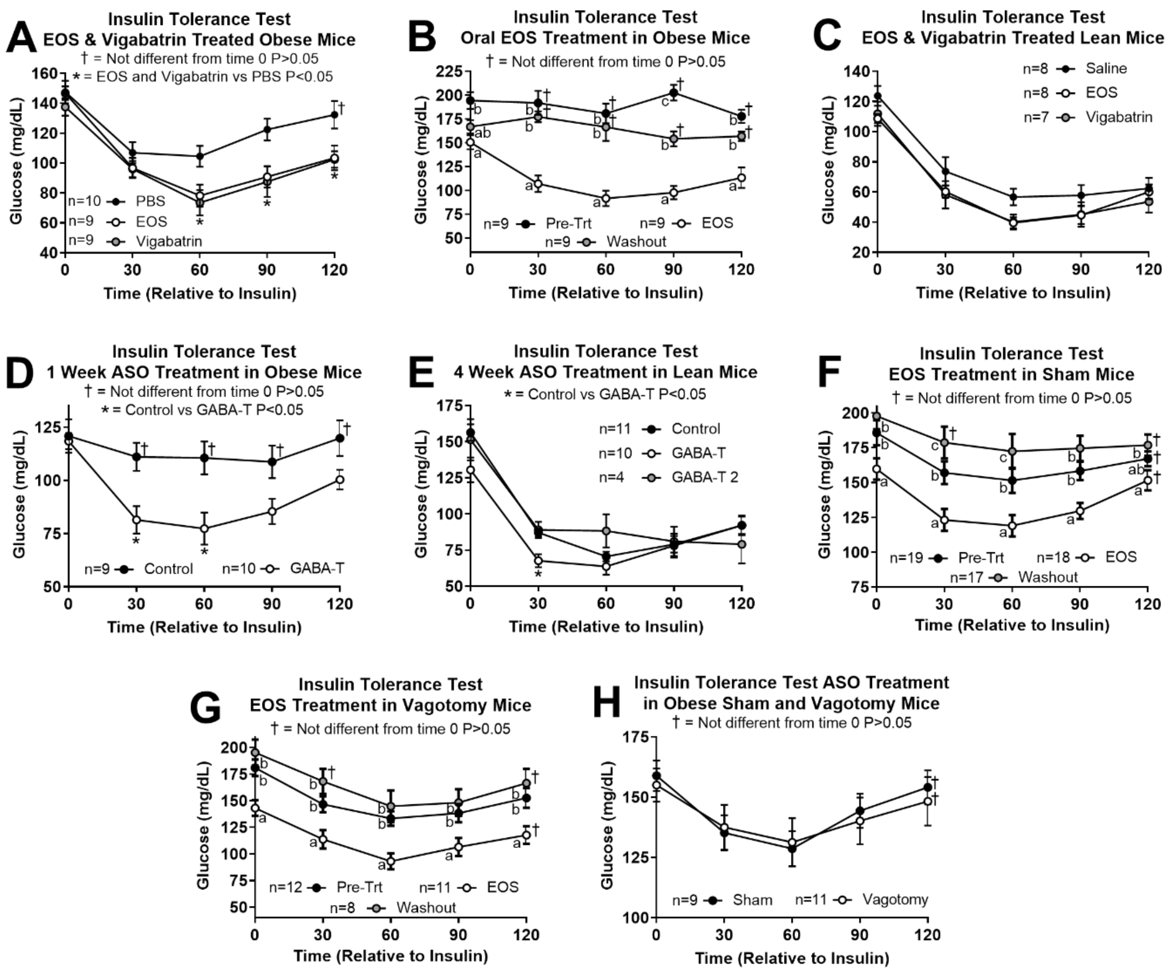


**Figure S9.** Related to Figures 1 and 2. Insulin tolerance tests (ITT) presented as raw glucose values. ITT on day 4 of EOS or Vigabatrin (8 mg/day), or PBS treatment in obese mice (A). ITT pre-treatment, on day 4 of oral EOS (3 g/L in drinking water) treatment, and after a 2-week washout period (B). ITT on day 4 of EOS or Vigabatrin (8mg/day), or PBS treatment in lean mice (C). ITT in obese mice after 1 week of control or GABA-T antisense oligonucleotide (ASO) treatment (D). ITT in lean mice after 4 weeks of control, GABA-T, or GABA-T 2 ASO treatment (E). ITT in sham (F) and vagotomized mice (G) at pre-treatment, on day 5 of EOS (8 mg/day) treatment, and after a 2-week washout period. ITT in obese sham and vagotomized mice after 4 weeks of GABA-T ASO treatment (H). [a,b,c] data points that do not share a common letter differ significantly ($P < 0.05$) within a timepoint. † Denotes the data point is not significantly different from time 0 for that group ($P > 0.05$). Unless indicated, all other timepoints are significantly different from time 0 within a group of mice. * Denotes significance between groups specified in the panel within a timepoint. All data are presented as mean ± SEM.

**Supplemental Tables and Legends**

**Table S1.** Metabolic characteristics of the study subjects (n=19). Related to Figure 6.

| | Mean ± SEM | Range |
|---|---|---|
| Body mass index (kg/m$^2$) | 45.1 ± 1.3 | 35.9 - 55.6 |
| Intrahepatic triglyceride content (%) | 11.4 ± 1.9 | 2.7 - 28.0 |
| Glucose (mg/dL) | 97 ± 2 | 81 - 121 |
| Insulin (μU/mL) | 24.1 ± 1.7 | 13.1 - 46.5 |
| Glucose infusion rate during insulin infusion (μmol/kg FFM/min) | 36.0 ± 3.0 | 15.2 - 60.8 |
| Glucose Rd during insulin infusion (% increase) | 131 ± 19 | 30 - 355 |

FFM, fat free mass; Glucose Rd, glucose disposal rate.

**Table S2.** Related to Figure 6. Regression coefficient estimates showing the association between hepatic mRNA expression of genes involved in GABA production (*ABAT*) and GABA transport (*SLC6A6, SLC6A8, SLC6A12, and SLC6A13*) and basal plasma insulin concentration (μU/mL) or hepatic insulin sensitivity index (HISI).

| **Basal Plasma Insulin Concentration (μU/mL)** | | | | | |
|---|---|---|---|---|---|
| | **Estimate** | **SEM** | **Lower CI** | **Upper CI** | **P- Value** |
| **Intercept** | -52.50 | 40.31 | -143.67 | 38.68 | 0.2251 |
| **IHTG (%)** | 0.30 | 0.14 | -0.01 | 0.62 | 0.0577 |
| ***ABAT*** | 18.14 | 5.28 | 6.19 | 30.09 | 0.0075 |
| ***SLC6A12*** | -14.71 | 4.29 | -24.41 | -5.01 | 0.0075 |
| ***SLC6A13*** | 1.90 | 3.14 | -5.19 | 8.99 | 0.5595 |
| ***SLC6A6*** | 3.02 | 2.40 | -2.41 | 8.45 | 0.2395 |
| ***SLC6A8*** | -1.87 | 1.47 | -5.18 | 1.45 | 0.2342 |
| | | | | | |
| **HISI** | | | | | |
| | **Estimate** | **SEM** | **Lower CI** | **Upper CI** | **P- Value** |
| **Intercept** | 16.41 | 7.23 | 0.06 | 32.76 | 0.0493 |
| **IHTG (%)** | -0.05 | 0.03 | -0.11 | 0.00 | 0.0674 |
| ***ABAT*** | -2.94 | 0.95 | -5.08 | -0.80 | 0.0127 |
| ***SLC6A12*** | 2.38 | 0.77 | 0.64 | 4.12 | 0.0127 |
| ***SLC6A13*** | -0.71 | 0.56 | -1.98 | 0.56 | 0.2379 |
| ***SLC6A6*** | -0.21 | 0.43 | -1.19 | 0.76 | 0.6340 |
| ***SLC6A8*** | 0.17 | 0.26 | -0.43 | 0.76 | 0.5416 |

**Table S3.** Related to Figure 6. Single Nucleotide Polymorphisms (SNPs) in the promoter of the ABAT gene, which encodes for GABA transaminase, are associated with a decreased odds ratio (OR) for type 2 diabetes (T2D; Source: knowledge portal diabetes database). MAF – minor allele frequency.

| ***ABAT* - T2D Associated SNPs** | | | | | | | |
|---|---|---|---|---|---|---|---|
| **Variant ID** | **dbSNP ID** | **Predicted Impact** | **Study** | **P-value** | **Effect** | **OR** | **MAF** |
| 16_8743360_C_G | rs72768103 | Promotor-intergenic | 70KforT2D GWAS | 0.00792 | ↓ | 0.872 | 0.007-0.036 |
| 16_8762951_G_A | rs185391944 | Promotor-intergenic | DIAGRAM 1000G GWAS | 0.0036 | ↓ | 0.852 | 0.005-0.01 |
| 16_8758576_C_G | rs12933032 | Promotor-intergenic | UK Biobank T2D GWAS (DIAMANTE-Europeans Sept 2018) | 0.028 | ↓ | 0.962 | 0.1 |

**Table S4.** Related to Figure 6. Single Nucleotide Polymorphisms (SNPs) that result in missense mutations in GABA transporters are associated with BMI (Source: knowledge portal diabetes database). MAF – minor allele frequency.

| *SLC6A12* - BMI Associated SNPs | | | | | | | |
|---|---|---|---|---|---|---|---|
| **Variant ID** | **dbSNP ID** | **Predicted Impact** | **Study** | **P-value** | **Effect** | **Effect Size** | **MAF** |
| 12_313824_G_A | rs199521597 | Missense: Replaces Alanine with Valine | GIANT 2018 BMI, Height exome chip analysis: African Americans | 0.0026 | ↑ | 3 | Not Reported |
| 12_319125_A_G | rs557881 | Missense: Replaces Cysteine with Arginine | GIANT UK Biobank GWAS | 0.0031 | ↑ | 0.0053 | 0.426 |
| 12_309921_T_C | rs143648821 | Missense: Replaces Isoleucine with Valine | FUSION exome chip analysis | 0.00316 | ↑ | 0.815 | 0.00105 |
| 12_309864_C_T | rs11061915 | Missense: Replaces Valine with Isoleucine | 13K exome sequence analysis | 0.00623 | ↑ | 0.299 | 0.00354 |
| 12_300248_C_G,T | rs147574089 | Missense: Replaces Glutamate with Glutamine | GIANT 2018 BMI, Height exome chip analysis: Hispanics | 0.0081 | ↑ | 0.65 | NaN |
| 12_300298_C_T | rs537332809 | Missense: Replaces Arginine with Glutamine | 13K exome sequence analysis | 0.0141 | ↑ | 1.09 | 0.000381 |
| | | | | | | | |
| *SLC6A6* - BMI Associated SNPs | | | | | | | |
| **Variant ID** | **dbSNP ID** | **Predicted Impact** | **Study** | **P-value** | **Effect** | **Effect Size** | **MAF** |
| 3_14523209_G_A | rs141254266 | Missense: Replaces Valine with Isoleucine | 13K exome sequence analysis | 0.00922 | ↑ | 0.866 | 0.00009-0.0003 |
| 3_14523296_G_A | rs41284017 | Missense: Replaces Valine with Isoleucine | GIANT 2018 BMI, Height exome chip analysis | 0.016 | ↑ | 0.028 | 0.004-0.0165 |
| 3_14526454_G_A | rs200063855 | Missense: Replaces Arginine with Histadine | GIANT 2018 BMI, Height exome chip analysis: South Asians | 0.033 | ↓ | -0.71 | 0.00007-0.0002 |
| | | | | | | | |
| *SLC6A13* - BMI Associated SNPs | | | | | | | |
| **Variant ID** | **dbSNP ID** | **Predicted Impact** | **Study** | **P-value** | **Effect** | **Effect Size** | **MAF** |
| 12_332337_C_T | rs202217743 | Missense: Replaces Valine with Leucine, or Methionine | FinnMetSeq exome sequence analysis | 0.0123 | ↑ | 1.26 | 0.0001 |
| 12_331781_C_T,G | rs147388541 | Missense: Replaces Aspartate with Histadine, or Asparginine | GIANT 2018 BMI, Height exome chip analysis: South Asians | 0.015 | ↑ | 0.59 | 0.003-0.02 |
| 12_347102_C_T | rs138506621 | Missense: Replaces Glutamate with Lysine | GIANT 2018 BMI, Height exome chip analysis: East Asians | 0.034 | ↑ | 2.1 | 0.00014-0.001 |
| 12_352884_C_T | rs543043546 | Missense: Replaces Glycine with Serine | 13K exome sequence analysis | 0.0341 | ↑ | 0.489 | 0.00001-0.00055 |
